# Functional characterization in *Chimonobambusa utilis* reveals the role of *bHLH* gene family in bamboo sheath color variation

**DOI:** 10.3389/fpls.2025.1514703

**Published:** 2025-02-12

**Authors:** Long Tong, Qingping Zeng, Yuan Guo, Yanjie Li, Hongyan Li, Lijie Chen, Xia Liu

**Affiliations:** ^1^ College of Smart Agriculture, Chongqing University of Arts and Sciences, Chongqing, China; ^2^ Bamboo Research Institute, Chongqing Academy of Forestry, Chongqing, China; ^3^ International Centre for Bamboo and Rattan, Chinese Academy of Forestry, Beijing, China; ^4^ Research Institute of Subtropical Forestry, Chinese Academy of Forestry, Hangzhou, China

**Keywords:** *Chimonobambusa utilis*, anthocyanin, bHLH gene family, qRT-PCR, protein interaction network

## Abstract

**Introduction:**

The basic helix-loop-helix (bHLH) proteins are a large family of transcription factors that are essential to physiology, metabolism, and development. However, the available information is limited about the *bHLH* gene family in *Chimonobambusa utilis*, which is widely cultivated in China because of its high-quality and economic value. *C. utilis* cultivars exhibit five natural color variations in their shoot sheaths, but the molecular mechanism behind this color diversity remains unclear.

**Methods:**

*De novo* assembly was employed to obtain gene sequences. To identify pathways related to color formation, GO enrichment analysis was performed on the 44,255 functionally annotated unigenes.

**Results:**

The transcriptomic analysis of *C. utilis* yielded a total of 195,977 transcripts and 75,137 unigenes after removing redundancy. The enrichment results revealed that four pathways were most strongly associated with color formation. Phylogenetic, conserved motif, and protein–protein interaction analyses, along with qRT–PCR validation, confirmed *CubHLH17*'s role in red sheath color.

**Discussion:**

This research not only deepens insights into the functional roles of *CubHLH* genes but also lays the foundation for genetic improvement of bamboo species. We suggest that these findings will contribute to both scientific research and commercial bamboo cultivation through gene editing technology in the future.

## 1 Introduction

China has rich bamboo germplasm resources and a large bamboo industry with more than 500 species and the bamboo forest area of more than 7 million hectares. China is a major producer and exporter of bamboo shoots (Yu et al., 2023). *Chimonobambusa utilis* (Keng) P.C. Keng (family Poaceae), a famous bamboo species having high economic value, is mainly distributed in the Jinfo Mountain (Nanchuan district, Chongqing) of China. This species has gradually formed a population with five different shoot sheath colors and relatively stable genetic traits during a long evolutionary process (Yang et al., 2022). *C. utilis* bamboo shoots, being tasty and crisp, and having rich fiber, high amino acid content, and vibrant colors, are known as the “king of bamboo shoots.” Because of their high quality, these shoots are preferred by consumers in both domestic and foreign lands (Chen et al., 2019; Yang et al., 2022). Bamboo shoot sheaths, a distinguishing characteristic of bamboo species, protect the young bamboo shoots and significantly influence their growth and quality. The color of bamboo shoots is a key agronomic characteristic that directly affects price and market competitiveness (Wang et al., 2020; Chen et al., 2021a; Yu et al., 2023).

The basic helix–loop–helix (bHLH) transcription factors constitute the second largest family in plants, and together with MYB and WD40, they regulate the biosynthesis of anthocyanins (Yu et al., 2024; Zhai et al., 2024) and participate in diverse physiological and biochemical processes in plants, including secondary metabolite biosynthesis, signal transduction, growth and development, and responses to biotic and abiotic stresses (Makkena and Lamb, 2013; Song et al., 2021; Chen et al., 2017). The bHLH domain consists of approximately 50–60 amino acids with two functionally distinct regions: a basic region and a HLH region (Ke et al., 2020). The basic region, located at the N-terminus, comprises approximately 17 amino acids (Murre et al., 1989; Feller et al., 2011). The HLH region includes two amphipathic helices made up of hydrophobic residues connected by a variable loop, which serves as a dimerization domain (Alsamman et al., 2023). Anthocyanins are pigments that determine the color of plant leaves or flowers (Streisfeld and Kohn, 2005; Cooley and Willis, 2009). The bHLH transcription factor was first discovered in maize, where it was found to be involved in anthocyanin biosynthesis (Ludwig et al., 1989). Subsequent studies have reported that bHLH transcription factors are involved in the biosynthesis of anthocyanin in various plants such as cherry (Wang et al., 2019), kiwifruit (Li, 2021), grape (Li et al., 2021a), colored-leaf poplar (*Populus deltoides*) (Wang et al., 2022), and pear (Han et al., 2023). Four *CcBHLH* genes in *Cinnamomum camphora* were identified as potentially involved in anthocyanin biosynthesis (Gong et al., 2023). The *CmBHLH2* gene was identified in chrysanthemum and is positively correlated with anthocyanin content, and its upregulation of *CmDFR* promotes anthocyanin accumulation (Xiang et al., 2015). Although the *bHLH* gene family has been reported in several species, it has not yet been studied in bamboo (*C. utilis*).

High-throughput sequencing technologies and a growing number of molecular tools have made it possible to accurately identify genes associated with plant organ coloration and to understand the developmental mechanisms in detail. In this study, we employed *de novo* assembly to obtain the gene sequences of *C. utilis*. Gene Ontology (GO) enrichment analysis was performed on all unigenes, and genes enriched in color-related pathways were selected for differential expression analysis. Potential *CubHLH* candidates associated with color variation were identified based on transcriptome data analysis. Moreover, the physicochemical properties, protein structures, subcellular localization, protein–protein interaction network, and phylogenetic relationships of the *bHLH* family members were analyzed. Finally, candidate *CubHLH17* genes related to bamboo sheath color variation were identified via qRT-PCR. These studies not only increase our understanding of the functions of the *CubHLH* gene family but also provide important scientific insights for bamboo genetic improvement and industrial applications, such as aesthetic appeal, quality, and market value, thereby contributing to both scientific research and commercial bamboo cultivation.

## 2 Materials and methods

### 2.1 Plant materials and RNA-seq

We selected *C. utilis*, which has five sheath colors: red, green, brown, yellow, and black. For ease of description, we simplified the cultivar names and used the following abbreviations: black-sheath cultivar, Bsh; brown-sheath cultivar, Brsh; red-sheath cultivar, Rsh; green-sheath cultivar, Gsh; and yellow-sheath cultivar, Ysh. Bamboo sheath samples were collected from Jinfo Mountain, Nanchuan District, Chongqing, China (29.012040° N, 107.195879° E), where the plant grows at an altitude of 2,129 m. On 19 September 2022, bamboo shoots of varying sheath colors were collected approximately 20 cm above the ground. The outermost layer of the sheath was removed, and the sheath material was cut approximately 5 cm from the tip of the bamboo shoot with scissors. The five bamboo sheath colors of *C. utilis* were collected with three biological replicates (15 samples in total). Fresh sheaths sampled for transcriptome sequencing quickly placed in liquid nitrogen containers and then stored in the laboratory at −80°C until RNA extraction. Total RNA was extracted using TRIzol reagent (Invitrogen, Waltham, USA). The purity of total RNA was assessed using electrophoresis in a 1.2% agarose gel and then verified based on absorbance values (260 nm/280 nm >1.8) using a Nanodrop 2000 Instrument (Thermo Fisher Scientific Inc., USA). An Agilent 2100 bioanalyzer (Agilent Technologies, Palo Alto, CA, USA) was used to assess RNA integrity. High-quality RNAs were subsequently subjected to high-throughput sequencing on the Illumina HiSeq 2500 platform.

### 2.2 *De novo* transcriptome assembly

The NGS QC Toolkit (http://www.nipgr.res.in/ngsqctoolkit.html) (Patel and Jain, 2012) was utilized to filter the raw data before assembly, and the adaptor sequences, reads with N ratios greater than 5%, and low-quality reads were removed. Finally, clean, high-quality reads were obtained. Trinity software v2.11.0 (Grabherr et al., 2011) was used for *de novo* assembly to generate transcripts and unigenes. Functional annotations and protein domain predictions were performed by aligning against the Nr, Swiss-Prot, GO, COG/KOG, KEGG, and Pfam databases using BLASTP and HMMER v3.3.2 (Mistry et al., 2013). Pearson correlation coefficients were calculated to assess the repetitiveness among samples, and a correlation heatmap was generated.

### 2.3 GO enrichment, differential gene expression analysis, and conserved motif analysis of the *CubHLHs*

Gene Ontology (GO) enrichment analysis of the unigenes was performed using the GO database (http://www.geneontology.org). GO enrichment analysis was conducted using the clusterProfiler v4.0 R package (Wu et al., 2021). The significance of each GO term was evaluated using Fisher’s test, and the p-value was adjusted for multiple hypothesis testing with the Benjamini−Hochberg method. GO terms with an adjusted p-value ≤ 0.05 were considered significantly enriched. WEGO software (Ye et al., 2006) was used to visualize the GO classifications of all unigenes. The DESeq2 R package v1.16.1 (Love et al., 2014) was used to analyze differential expression between the two groups, with differentially expressed genes identified based on FDR-adjusted p-value < 0.01 and log2-fold change ≥ 2. Conserved motif analysis of the *CubHLH* gene family was conducted using the online tool MEME (https://meme-suite.org/meme/), and the motifs were visualized in TBtools (Chen et al., 2020).

### 2.4 Identification of the *CubHLH* gene family

We downloaded the sequence of bHLH proteins in *Arabidopsis* from the PlantTFDB v4.0 database (Jin et al., 2017). The profile hidden Markov models (HMMs) of the DNA-binding domains for the *bHLH* gene family (PF00010) were downloaded from the Pfam database (http://pfam-legacy.xfam.org/) and searched against the *C. utilis* protein sequence using HMMER (Finn et al., 2011) to identify candidate *bHLH* genes of *C. utilis*. The E-value cutoff was set to 10^−5^. To verify the reliability of the BLAST and HMMER intersection results, the completeness of the candidate gene domains was then checked using the Conserved Domain Database (CDD) from NCBI (https://www.ncbi.nlm.nih.gov/).

### 2.5 Physicochemical properties and subcellular localization prediction of the *CubHLH* gene family

The physicochemical properties of the *CubHLH* gene family, including amino acid number, molecular weight, and isoelectric point were analyzed using TBtools (Chen et al., 2020). The subcellular localization of the *CubHLH* gene family was predicted using the Plant mPLoc tool (http://www.csbio.sjtu.edu.cn/bioinf/plant-multi/). Additionally, the secondary and tertiary structures of the *C. utilis* bHLH family proteins were predicted using the SOPMA online tool (https://npsa-prabi.ibcp.fr/cgi-bin/npsa_automat.pl?page=npsa_sopma.html).

### 2.6 Sequence alignment, phylogenetic relationships, and prediction of the protein–protein interaction network of *CubHLHs*

Sequence alignment was carried out with MUSCLE. A phylogenetic tree of the *CubHLH* gene family was constructed using MEGA v7.0 (Hall, 2013) with the maximum likelihood (ML) method, and bootstrap analysis was set to 1,000 replicates. The protein–protein interaction networks were constructed for the identified *CubHLH* candidate genes using the online tool STRING v11.5 (https://string-db.org/), with *A. thaliana* selected as the reference species model and a confidence interaction score ≥0.7.

### 2.7 Expression profile analysis of *CubHLHs*

The RNA sequencing reads were aligned to the unigene library using Bowtie2 v2.4.4. Using the R script, the average expression of *C. utilis* genes in different color bamboo shoot sheaths was calculated based on normalized expression (fragments per kilobase per million mapped reads, FPKM), and RSEM (Li and Dewey, 2011) was used to estimate gene expression levels and then obtain the FPKM values. A heatmap was created using TBtools (Chen et al., 2020).

### 2.8 qRT-PCR analysis

Complementary DNA (cDNA) was synthesized using the Synthesis SuperMix for qPCR (One-Step gDNA Remover) Kit (Wuhan Servicebio Technology Co., Ltd., Wuhan, China). qRT-PCR was performed using 2×Universal Blue SYBR Green qPCR Master Mix (Wuhan Servicebio Technology Co., Ltd., Wuhan, China) in a 20-μL reaction volume on a CFX Connect Real-Time PCR Detection System (Bio-Rad). The primers were designed using Primer v5.0 software. The *TIP41* (Tonoplasm Intrinsic Protein 41) gene was used as an internal reference gene and was obtained from a previous report (Fan et al., 2013). **Supplementary Table S1** lists the primers used in qRT-PCR. The 2^−ΔΔCT^ method (Livak and Schmittgen, 2001) was employed to calculate the relative expression level of the candidate gene, and the expression levels of green bamboo shoot sheaths were used as the reference control. All qRT-PCR experiments were conducted with three biological replicates.

### 2.9 Statistical analysis

Statistical analysis of the bamboo sheath samples was performed in Excel 2021 using Student’s *t*-tests (*p* < 0.05). Statistical significance was determined via GraphPad Prism v9.5.1, and the data are presented as the mean ± standard error (SE). GraphPad Prism was used to generate the figures.

## 3 Results

### 3.1 RNA-Seq analysis and *de novo* assembly

**Figure 1:**
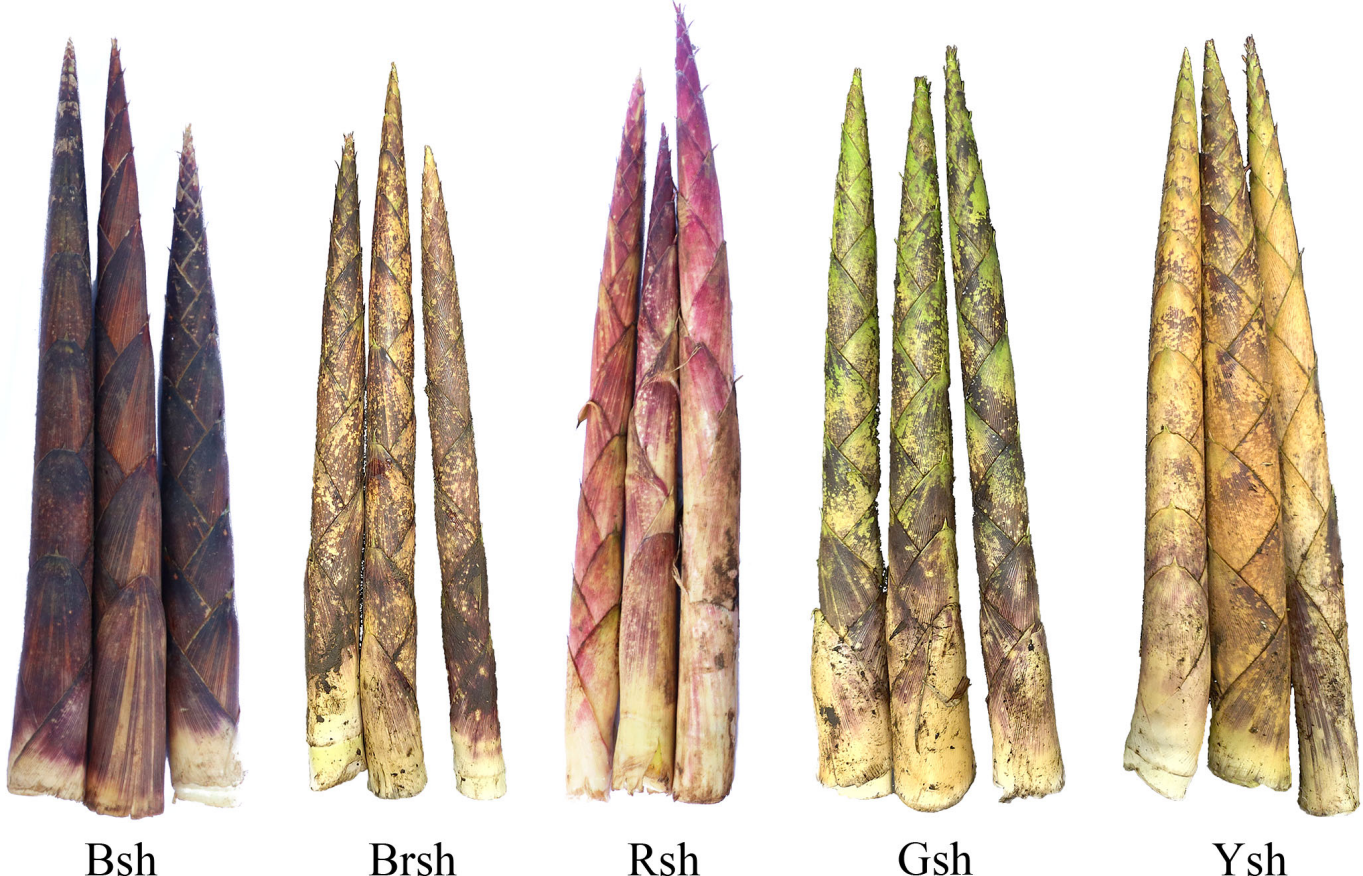
The phenotypes of different shoot sheaths color in *Chimonobambusa utilis*. Bsh represents black sheaths, Brsh represents brown sheaths, Rsh represents red sheaths, Gsh represents green sheaths, and Ysh represents yellow sheaths.

Upon visual inspection, the color differences among the five bamboo varieties were obvious (**Figure 1**). High-throughput sequencing was performed with three biological replicates for each of the 15 samples. A total of 131.38 Gb of clean data were obtained, with at least 7.68 Gb per sample. The GC content ranged between 46% and 49%, with no N bases detected, indicating data integrity (**Supplementary Table S2**). The Q20 values for all samples exceeded 96%, the Q30 base percentage was 91.27% or higher, and the cycle Q20 reached 100% in each sample, indicating excellent sequencing quality and minimal impact from sequencing cycles. After *de novo* assembly using high-quality reads, we obtained 195,977 transcripts and 75,137 unigenes after removing redundancy. The N50 value is an important indicator of assembly quality, reflecting the length continuity and integrity of a sequence. The results of this study showed that the N50 value of the transcripts was 1,704, and the N50 value of the unigenes was 1,370, indicating good assembly quality and suitability for subsequent analysis.

The unigenes were comprehensively annotated using multiple databases, including Nr, Swiss-Prot, GO, COG, KOG, and KEGG. The results indicated that 44,255 unigenes were successfully annotated with functional information. Additionally, protein domains within these unigenes were identified via alignment using the Pfam database and HMMER software, providing an essential basis for further investigations into gene functions and metabolic pathways. Pearson’s correlation coefficients revealed that a strong correlation between biological replicates within each group and showed that the samples were highly reproducible, particularly the correlation coefficient for the black-sheath (Bsh) and the brown-sheath (Brsh) samples, which was close to 0.92 (**Supplementary Figure S1**), indicating a high degree of consistency among the samples. The correlation coefficients for the yellow-sheath (Ysh) and red-sheath (Rsh) samples were 0.85 and 0.88 (**Supplementary Figure S1**), respectively, further confirming the reliability of the data. No significant batch effects or experimental biases were observed.

### 3.2 GO enrichment analysis

**Figure 2:**
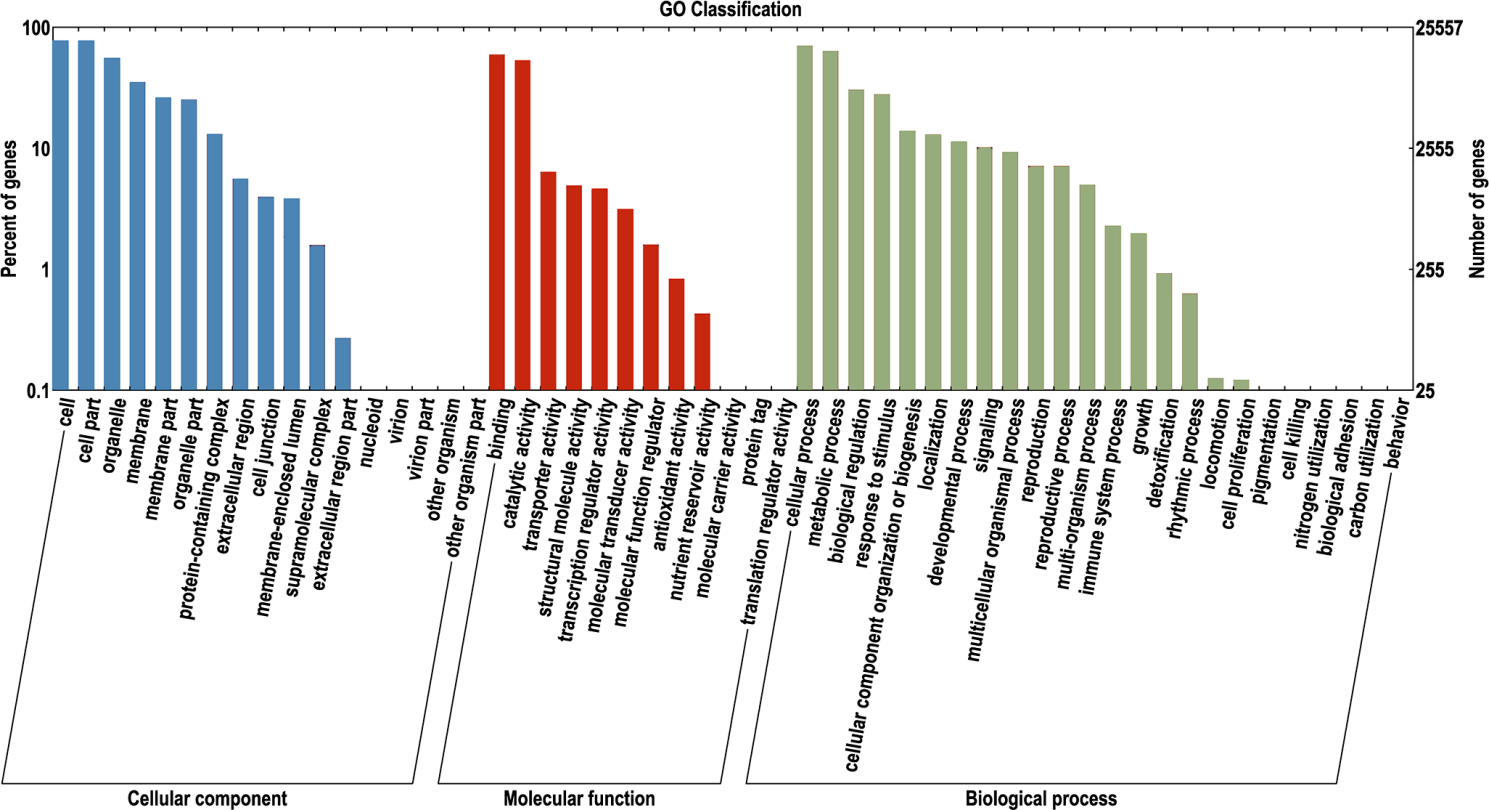
The results of GO enrichment analysis. The x-axis indicates the categories, with blue representing enrichment in cellular component, red representing enrichment in molecular function, and green representing enrichment in biological process. The y-axis represents the percentage of enriched items.

To identify pathways related to color, GO enrichment analysis was performed on the 44,255 functionally annotated unigenes. The enrichment results revealed that four pathways were most strongly associated with color (**Figure 2**). Pigmentation is a pathway that is directly related to color and involves the production, distribution, and deposition of pigments (Todesco et al., 2022). The transporter activity pathway is the site where pigment molecules are synthesized and accumulate. The genes in this pathway may be involved in regulating the spatial distribution of pigments (Wiriyasermkul et al., 2020). The third pathway, the catalytic activity pathway, may be enriched with genes encoding enzymes that catalyze pigment synthesis and are the core steps involved in regulating pigment synthesis (Zhang et al., 2022). Finally, the metabolic process pathway is enriched with genes related to the synthesis of secondary metabolites (such as flavonoids and anthocyanins), which determine color presentation (Li et al., 2021b). Focusing on the above core pathways, which included a total of 13,854 genes, we effectively located genes related to color.

### 3.3 Differential gene expression analysis based on GO

**Figure 3:**
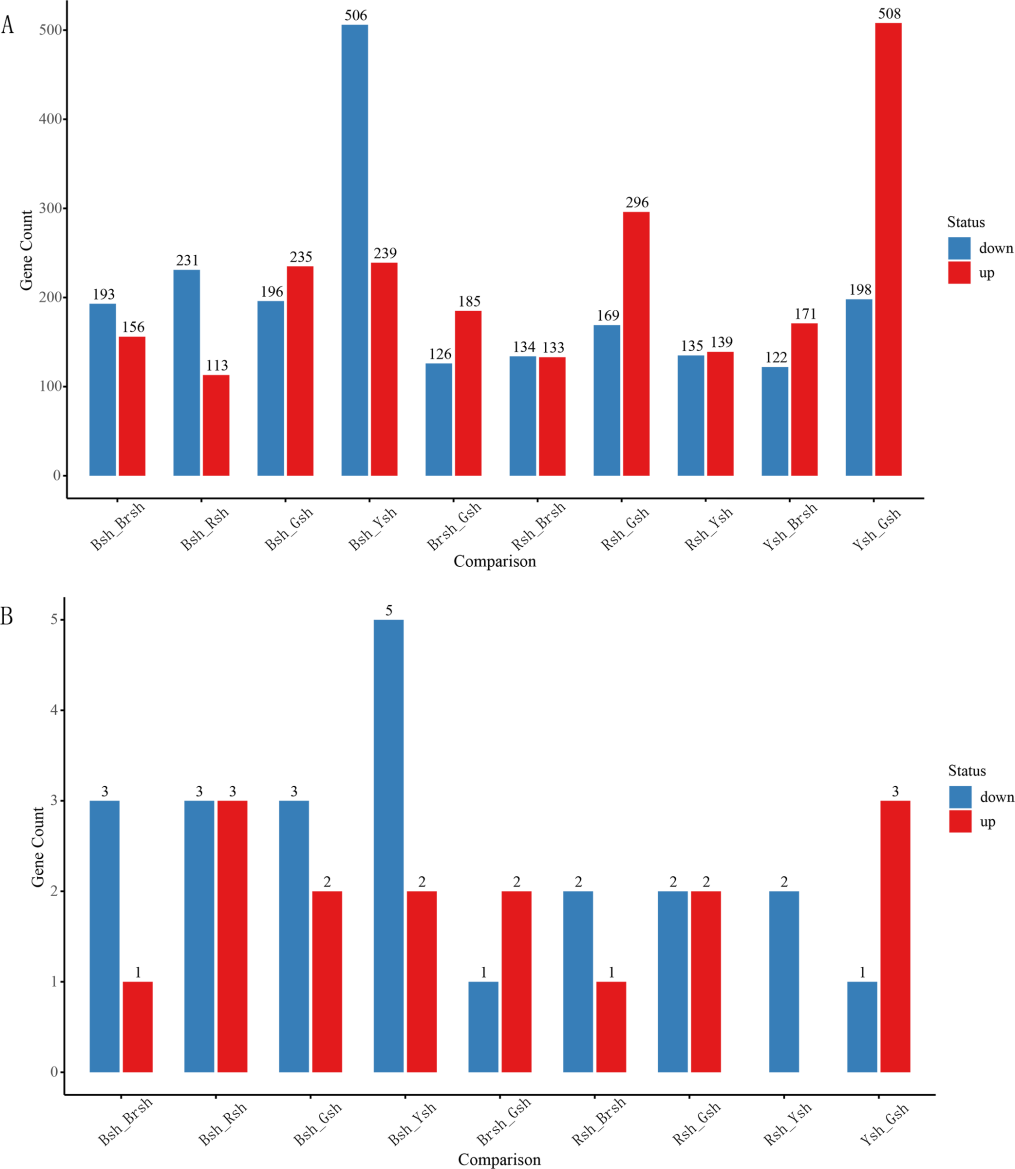
Differential gene analysis. **(A)** Visualization of the differential analysis based on the GO enrichment results of unigenes, where red indicates upregulation and blue indicates downregulation. The x-axis represents different groups in the differential analysis, and the y-axis represents the specific number of significant differential results. **(B)** Differential analysis of *CubHLH* gene family members, where red indicates upregulation and blue indicates downregulation. The x-axis represents different groups in the differential analysis, and the y-axis represents the specific number of significant differential results.

**Figure 4:**
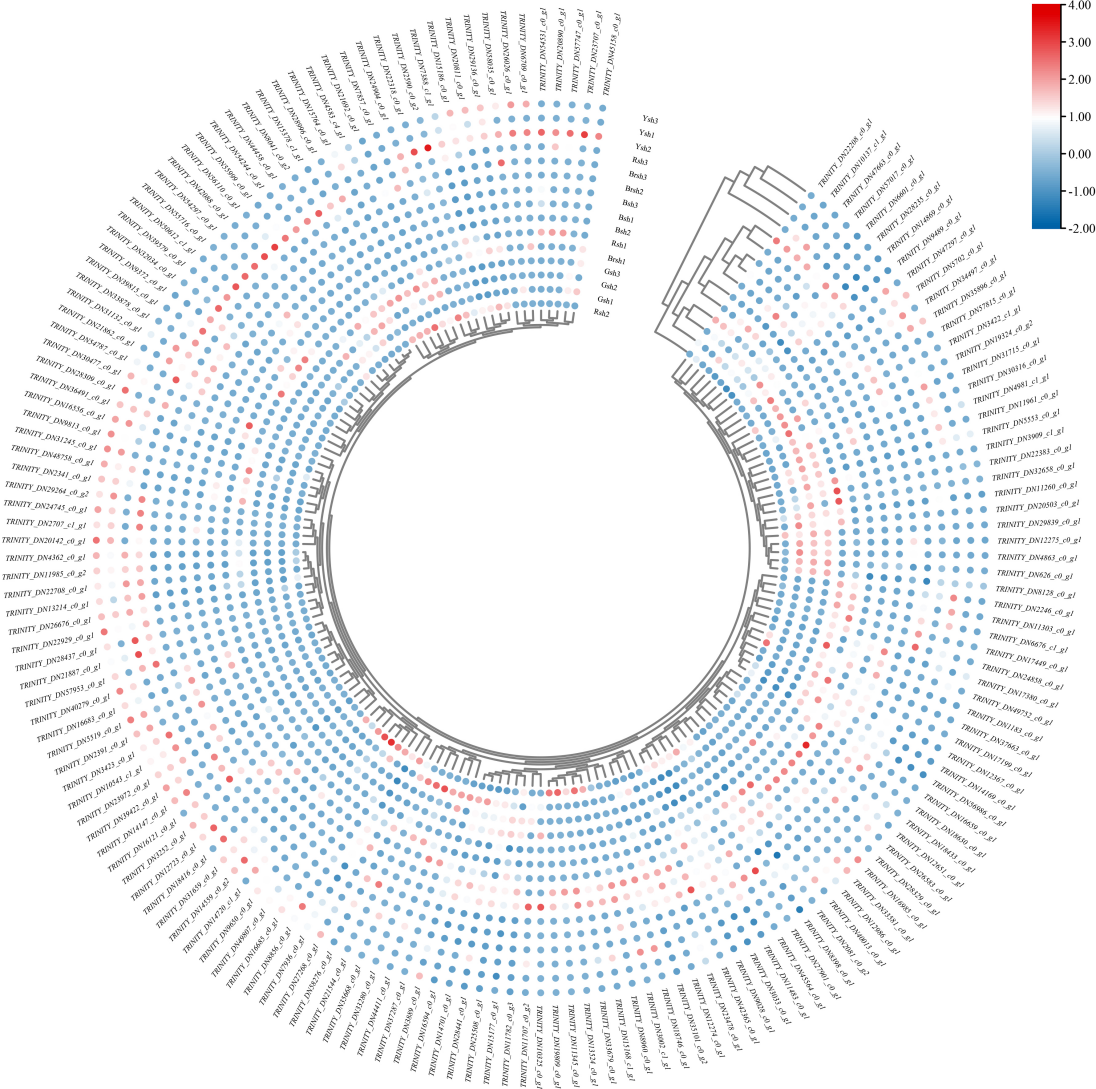
The heatmap of 168 significantly differentially expressed genes. The expression levels decrease gradually from red to blue. Each row represents the expression level of each gene across different samples. The scale bar indicates the log2-transformed FPKM values.

Differential expression analysis showed that a total of 4,185 genes were affected, of which 2,175 genes were upregulated and 2,010 genes were downregulated (**Figure 3A**). Further filtering of the differential gene expression analysis identified 168 significantly differentially expressed genes (**Figure 4**). There were obvious differences in the expression of these genes among the different samples. For example, *TRINITY_DN9650_c0_g1* showed extremely high expression levels in the Rsh sample, whereas expression was not detected in the Brsh group. It is speculated that this gene may be involved in the biosynthetic pathway or pigment formation mechanism related to the coloration of the Rsh group. The expression of the *TRINITY_DN4362_c0_g1* gene in the Ysh sample was significantly upregulated compared with that in the Gsh sample, indicating that it may play an important role in the biological processes or metabolic pathways of the Ysh group. Additionally, the expression of *TRINITY_DN23972_c0_g1* showed a similar trend, indicating its potential function in specific physiological activities of the Ysh sample. In contrast, some genes exhibited significant downregulation trends between different color groups. For example, *TRINITY_DN19324_c0_g2* showed high expression in the Gsh sample and almost no expression in the Rsh group, indicating that this gene may play a specific role in the biological process of the Gsh sample. The *TRINITY_DN22383_c0_g1* gene was significantly downregulated in the Rsh group compared with that of the Brsh group (**Figure 4**), further highlighting the considerable differences in gene expression between these different sheath color samples.

### 3.4 Identification, physicochemical property analysis, and differential expression analysis of the *CubHLH* gene family

Based on the protein database from the *C. utilis* transcriptome data, candidate genes were screened using the hmmbuild and hmmsearch tools in HMMER. The sequences were then submitted to NCBI for validation via batch CD-Search. After sequences with incomplete domains or those lacking the bHLH domain were manually removed, a total of 44 *CubHLH* gene family members were identified and designated *CubHLH1* to *CubHLH44* (**Supplementary Table S3**). The specific corresponding original IDs can be found in **Supplementary Table S4**. Physicochemical property analysis revealed that the amino acid lengths of the *CubHLH* gene family ranged from 100 to 618 amino acids, with molecular weights ranging from 10,777.15 to 67,522.22 Da (**Supplementary Table S5**). The isoelectric points (pIs) ranged from 4.61 to 10.46 (**Supplementary Table S5**). There were 30 proteins with pIs > 7 and 14 with pIs < 7, which indicates that 30 of these proteins are basic amino acids and 14 are acidic amino acids. The instability index ranged from 34.12 to 88.18, and the aliphatic index was between 52.96 and 103.7. The grand average of hydropathy values (GRAVY) of the *CubHLH* gene family was negative (**Supplementary Table S5**), indicating that the *Cub*HLH family proteins are hydrophilic. Subcellular localization prediction of *CubHLH* gene family members showed that, with the exception of CubHLH37, which is located in the chloroplast, the remaining 43 genes were all localized in the nucleus (**Supplementary Table S5**). Using the SOPMA online tool to predict the secondary structure of the *CubHLH* gene family proteins (**Supplementary Figure S2**) revealed that the α-helix content ranged from 16.98% to 42.5%, the extended strand content ranged from 1.8% to 15%, and the β-sheet content ranged from 0.75% to 11%, with CubHLH30 lacking a β-sheet. The random coil content ranged from 30.18% to 70.11% (**Supplementary Table S6**).

To investigate the potential role of the *bHLH* gene family in the color formation of *C. utilis* bamboo shoot sheaths, differential expression analysis was performed on all candidate genes. Differentially expressed genes were analyzed between bamboo shoot sheath samples of different colors (Bsh, Gsh, Brsh, Ysh, and Rsh). A pairwise comparison was conducted, resulting in a total of 10 groups (**Supplementary Table S7**; **Figure 3B**). The results showed that no significant differentially expressed genes were identified between the Ysh and Brsh groups. However, *CubHLH3*, *CubHLH4*, and *CubHLH27* may play a role in the positive regulation of pigment synthesis in different bamboo sheath color samples. Notably, the *CubHLH3* and *CubHLH4* genes were upregulated in multiple groups, whereas the *CubHLH27* gene was specifically upregulated in the Bsh samples (**Supplementary Table S7**) and is potentially related to the accumulation of black pigments. Conversely, the *CubHLH1*, *CubHLH2*, *CubHLH7*, *CubHLH22*, and *CubHLH41*genes, which are downregulated, may negatively regulate color expression by inhibiting certain pigment biosynthesis pathways. The *CubHLH7* gene was downregulated in multiple groups (**Supplementary Table S7**), suggesting that it may suppress the expression of nonblack pigments in black bamboo shoot sheath samples. In addition, *CubHLH31* and *CubHLH30* showed a dual pattern of upregulation and downregulation in different comparison groups (**Supplementary Table S7**). We speculate that these genes may balance the synthesis and degradation of pigments through complex tissue-specific or developmental stage-specific regulatory mechanisms, ultimately affecting the color diversity of the bamboo shoot sheath and the development of bamboo sheath color.

The results of the physicochemical property analysis revealed that most of the upregulated genes have isoelectric points primarily in the neutral to slightly acidic range, have moderate molecular weights, and possess negative hydropathy indices, indicating that they are hydrophilic proteins. These proteins likely exert their transcriptional regulatory functions by binding to other proteins or DNA. The *CubHLH7* gene, which is downregulated in multiple groups, was found to have a relatively high isoelectric point and is also a hydrophilic protein, which is consistent with its localization in the nucleus (**Supplementary Table S5**). Therefore, based on the differential expression and physicochemical property analyses, it can be inferred that the upregulated *CubHLH* gene family members promote pigment synthesis primarily through their transcriptional regulatory functions in the nucleus, whereas downregulated genes such as *CubHLH7* may negatively regulate color expression by inhibiting specific pigment biosynthesis pathways. These findings suggest that the *CubHLH* gene family plays a complex regulatory role in the development of shoot sheath color in bamboo.

### 3.5 Motif prediction and expression patterns of *CubHLH* genes

**Figure 5:**
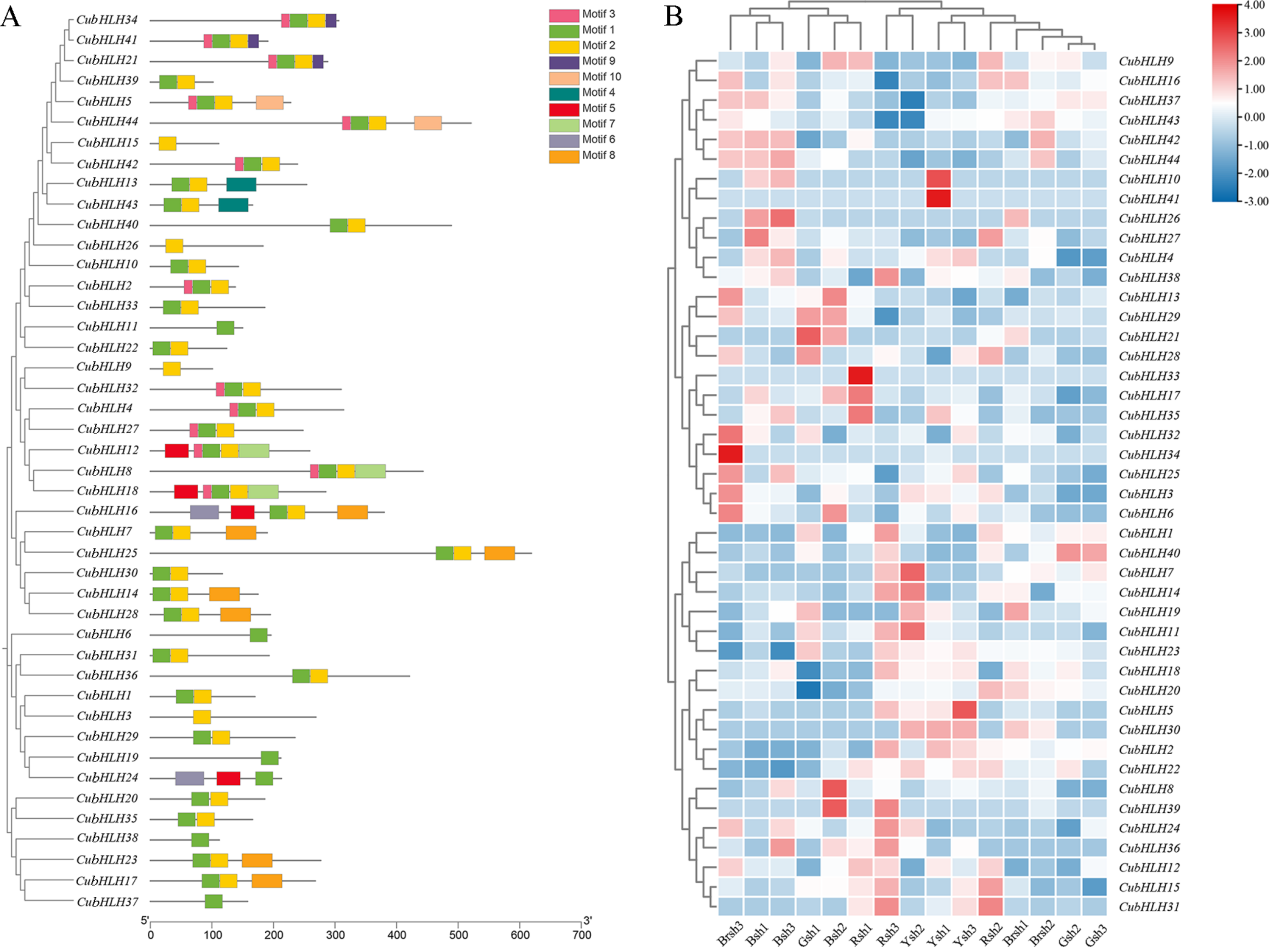
The predicted motif and expression profiles analysis of *CubHLHs*. **(A)** The predicted motifs for the candidate genes of the *CubHLH* gene family. The motifs are denoted by different colored boxes. **(B)** The heatmap of expression profiles of the *CubHLH* family candidate genes. The scale indicates the log2-transformed FPKM values, with a color spectrum from red to blue representing gene expression levels that range from high to low, respectively.

Conserved motif prediction and visualization analysis were performed on the 44 *CubHLH* gene family members (**Figure 5A**). A total of 10 motifs, designated motif1 to motif10, were identified. Each gene contained 1–5 motifs, among which motif 1 and motif 2 were the most numerous and were located primarily at the N-terminus (5′ end) of the protein. Specifically, motif 1 was present in 40 *CubHLH* genes, and motif 2 was found in 38 *CubHLH* genes. *CubHLH3*, *CubHLH9*, *CubHLH15*, and *CubHLH26* contained only motif 2, whereas *CubHLH6*, *CubHLH11*, *CubHLH19*, *CubHLH37*, and *CubHLH38* contained only motif 1. Some motifs existed only in specific genes, such as motif 4, which was present only in *CubHLH13* and *CubHLH43* of the vIIb subfamily. These specific motifs may confer unique functions to genes. Within the same evolutionary subgroup, most genes exhibited similar conserved motif compositions, suggesting that they may share similar biological functions. For example, *CubHLH21*, *CubHLH34*, and *CubHLH41* from the vIIIc subfamily all contained motifs 1, 2, 3, and 9, whereas *CubHLH17* from the IIIf subfamily contained motifs 1, 2, and 8. Given that the *CubHLH17* gene of the IIIf subfamily is closely related to anthocyanin biosynthesis, the conserved motif composition of this type of gene may be related to its function in pigment synthesis pathways.

The expression levels of the *CubHLH* genes were analyzed in bamboo shoot sheath samples of different colors, including black (Bsh1, Bsh2, and Bsh3), green (Gsh1, Gsh2, and Gsh3), brown (Brsh1, Brsh2, and Brsh3), yellow (Ysh1, Ysh2, and Ysh3), and red (Rsh1, Rsh2, and Rsh3) (**Figure 5B**). Clustering analysis of *CubHLH* gene expression levels among the samples revealed significant variation in expression levels between different samples for each gene.

### 3.6 Evolutionary analysis and the CubHLH protein–protein interaction network

**Figure 6:**
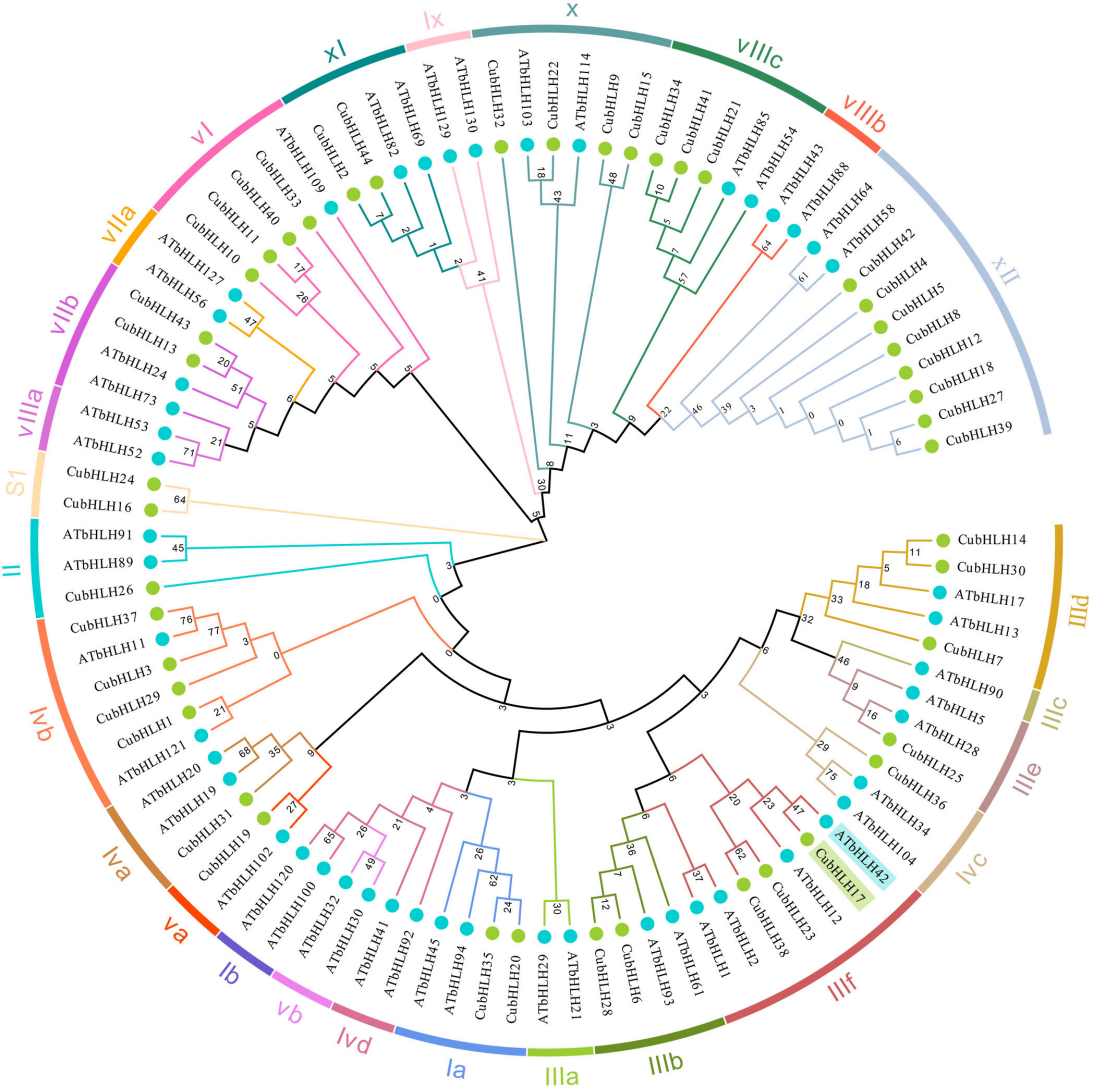
Phylogenetic tree analysis of *CubHLHs*. The 26 subgroups are marked in different colors on the periphery of the circle. The names of each subgroup, along with their respective gene IDs, are annotated around the outer perimeter of the tree for clarity. The blue circles represent the genes from the *Arabidopsis thaliana bHLH* family, and green circles represent the genes from the *C. utilis bHLH* family.

To explore the evolutionary relationships within the *CubHLH* gene family, a phylogenetic tree was constructed using the 44 CubHLH protein sequences and the protein sequences of *Arabidopsis* bHLH (**Figure 6**). Following the classification method of *Arabidopsis* (Heim et al., 2003; An et al., 2014), one to three representative genes from different subgroups were selected to construct a phylogenetic tree together with the *CubHLH* genes, resulting in a tree containing 49 *AtbHLH* and 44 *CubHLH* genes. These genes were ultimately classified into 26 subgroups. The results showed that the distribution of *CubHLHs* in the 26 subgroups was quite different, among which *CubHLH16* and *CubHLH24* belonged to the independent SI subfamily and did not cluster with any *Arabidopsis* group, which may reflect unique gene expansion or the generation of novel functions in *C. utilis*.

No corresponding *CubHLH* genes were found in some subgroups, such as Ib, Vb, Ivd, IIIa, IIIc, VIIIb, Ix, VIIa, and VIIIa, which may be the result of gene loss or evolutionary divergence. Notably, *CubHLH* genes were most abundant in subfamily XII, with a total of eight genes, whereas the remaining genes were more evenly distributed in other subgroups, with each group containing one to four genes. Prior research indicates that *AtbHLH12* and *AtbHLH42* in the IIIf subfamily are closely related to anthocyanin biosynthesis and that the accumulation of anthocyanins directly affects color expression in plants (Li et al., 2021a; Makkena and Lamb, 2013). Phylogenetic analysis showed that *CubHLH17* clustered with the IIIf subfamily, which shares the same branch as *AtbHLH42* (**Figure 6**), suggesting that *CubHLH17* may have functions similar to those of the homologous genes in *Arabidopsis thaliana* and could be involved in anthocyanin biosynthesis in *C. utilis* bamboo shoot sheaths.

**Figure 7:**
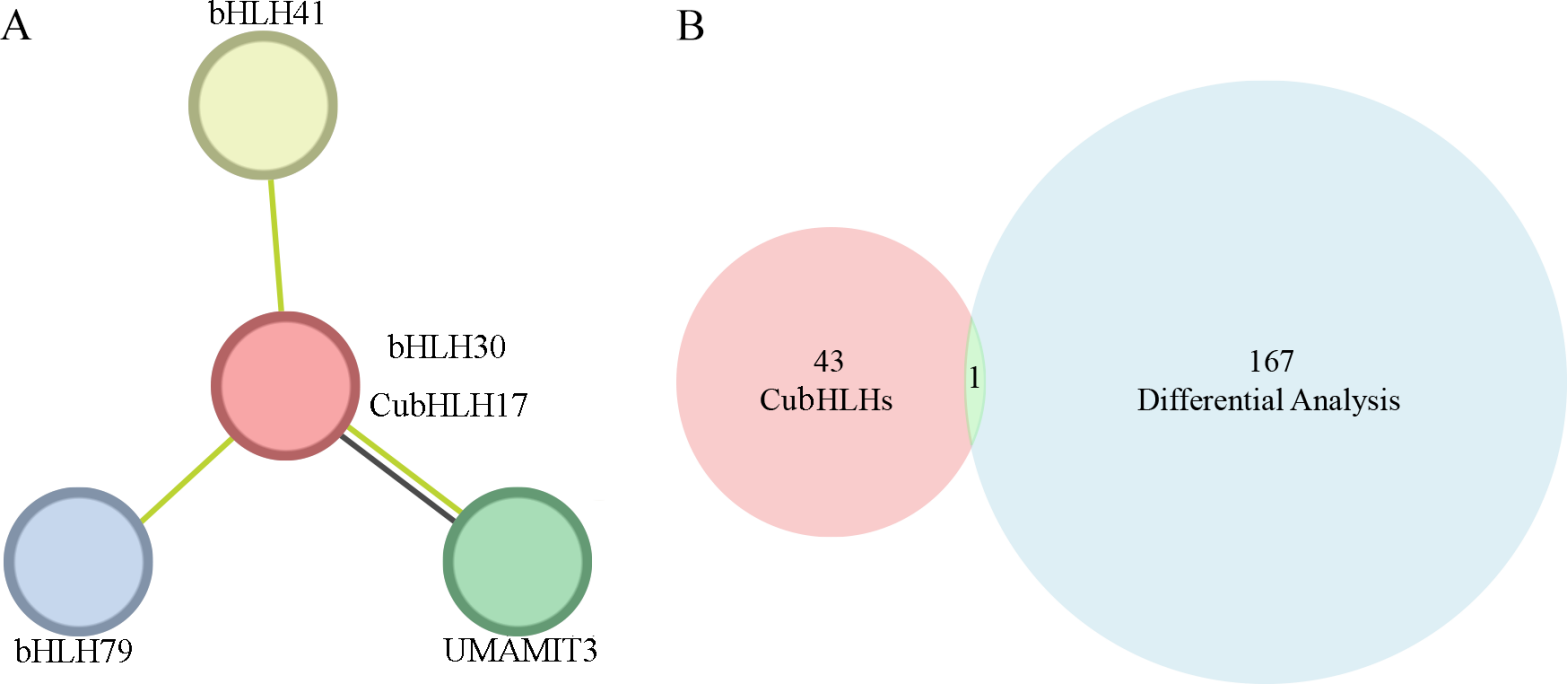
Protein–protein interaction network of CubHLH17 proteins in *C.*) *utilis* from the model plant *Arabidopsis thaliana* homologous genes. **(A)** Green lines represent associations proteins identified through text mining, and black lines represent co-expression relationships. The red circle in the center indicates that *bHLH30* is directly related to *CubHLH17* in the protein interaction network. **(B)** The Venn diagram depicts the overlap between two gene sets. The red circles showed the *CubHLH* gene family members, while the blue circles represent the genes identified through GO enrichment and differential expression analysis based on unigenes. The intersection in the middle represents the common genes identified by both datasets.

To further analyze the function of the *CubHLH* gene family in the coloration of *C. utilis*, this study selected *CubHLH17* gene, which is highly related to anthocyanin synthesis, for protein interaction network analysis. Using STRING for prediction and selecting *Arabidopsis* as the model organism, the results showed that *CuBHLH17* strongly interacts with four proteins and is the most closely associated with *bHLH30* (**Figure 7A**). A Venn diagram was drawn based on the 168 highly color-related genes identified via GO enrichment and the *CubHLH* gene family members (**Figure 7B**), and the results indicated that *CubHLH30* was identified by both approaches, implying that it may have some functions in the bamboo shoot sheath. The clustering of these proteins suggests that they may have similar or related functions. *CubHLH17* may participate in specific biological processes through interactions with these proteins, providing important clues for further functional studies.

### 3.7 Validation of the candidate gene *CubHLH17* by qRT-PCR

**Figure 8:**
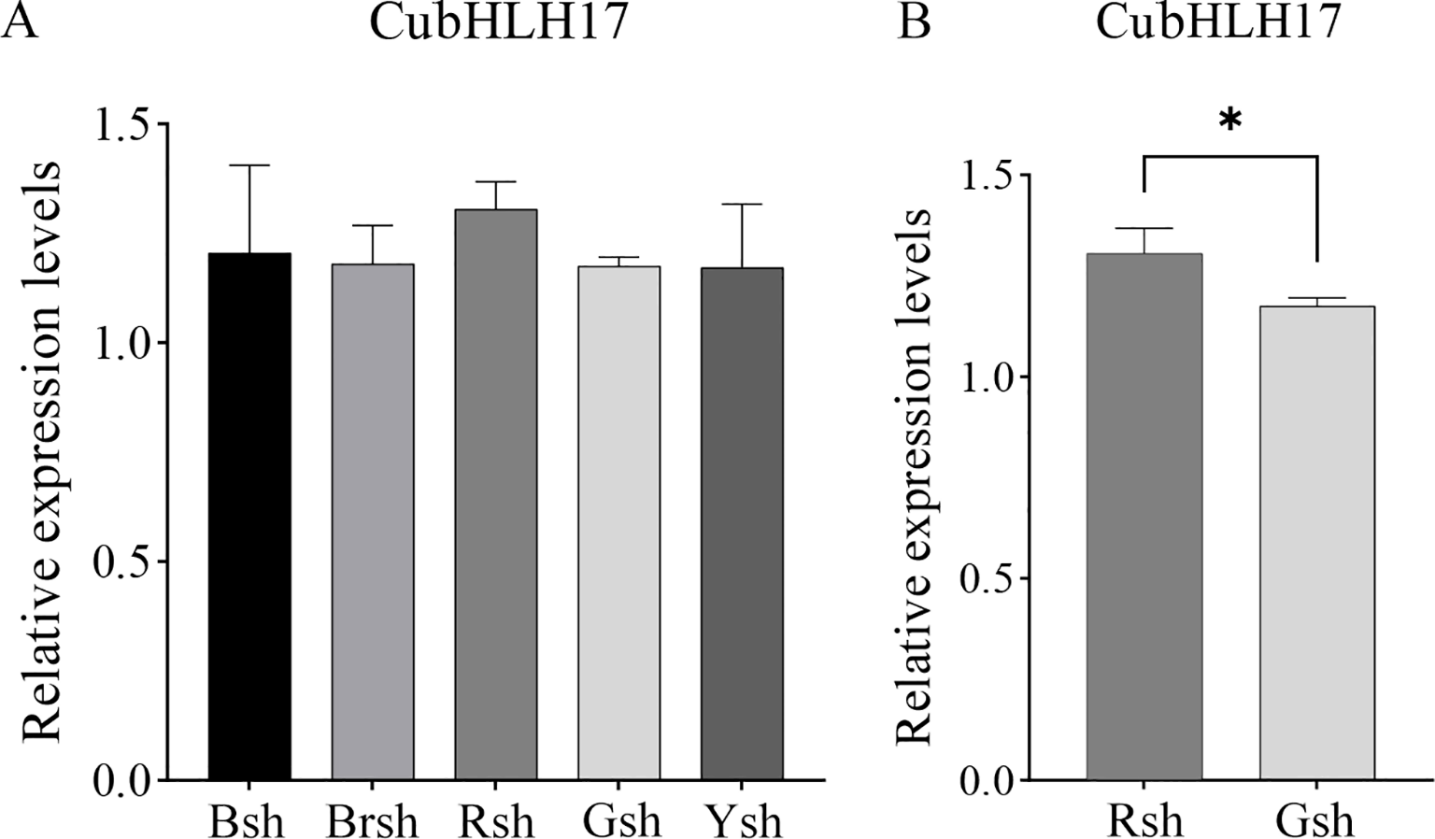
qRT-PCR analysis of the expression of transcription factor. **(A)** The relative expression levels of transcription factor coding gene of *CubHLH17*. Black bamboo sheath (Bsh), brown bamboo sheath (Brsh), green bamboo sheath (Gsh), red bamboo sheath (Rsh), and yellow bamboo sheath (Ysh). **(B)** The relative expression levels of *CubHLH17* between the Rsh and Gsh group. The internal reference gene was *TIP41*, the expression of green bamboo sheath as a reference. Bars are means of three replicates ± SE. Asterisks (*) indicate the statistical significance of the difference between the Rsh and Gsh group. (*p < 0.05).

Based on the results of the phylogenetic tree and protein interaction network analysis, we selected the color-related *CubHLH17* gene and used real-time quantitative qRT-PCR technology to analyze its expression in bamboo shoots of different colors (**Figure 8**). The results indicated that *CubHLH17* was expressed in all samples (**Figure 8A**), suggesting that it may play a regulatory role in the process of shoot sheath color formation in bamboo. A *t*-test revealed a significant difference in *CubHLH17* expression between the Rsh group and the Gsh group (**Figure 8B**). *CubHLH17* showed higher expression levels in Rsh, suggesting that *CubHLH17* may play a role in the development of specific colors. Protein−protein interaction analysis showed that *CubHLH17* is associated with *bHLH30* (**Figure 7A**), which is involved in red coloration. These results provide some evidence that *CubHLH17* may be involved in the regulation of red bamboo shoot sheath formation. In future studies, all color-related candidate genes identified in our study, along with *CubHLH17*, will be integrated with other omics approaches to further explore their key regulatory roles in the coloration of *C. utilis*.

## 4 Discussion

### 4.1 Potential regulatory factors in bamboo shoot sheath color formation

Elucidating the molecular mechanisms responsible for the coloration of bamboo shoot sheaths is essential for enhancing quality and market competition. Previous studies have shown that bHLH transcription factors play a key regulatory role in anthocyanin biosynthesis in plants (Zhao et al., 2021; Jiang et al., 2022; Liu et al., 2023). We identified 168 significantly differentially expressed genes based on *de novo* transcriptome assembly data and then selected the *bHLH* gene family related to coloration for analysis. A total of 44 *CubHLH* gene family members were identified in the shoot sheaths of *C. utilis*. Compared with the 602 *bHLH* genes identified in *Brassica napus* (Ke et al., 2020), 159 genes found in *Solanum lycopersicum* (Sun et al., 2015), 85 *bHLH* genes in *Ginkgo biloba* (Zhou et al., 2020), 120 *bHLH*s in *Camellia sinensis* (Cui et al., 2018), and 128 *bHLH* genes in *Betula platyphylla* (Zhao et al., 2023), the number of *bHLH* genes in *C. utilis* is relatively small. This difference may reflect the evolutionary characteristics of different species and the different degrees of gene family expansion. Differential expression analysis was performed on all candidate gene families, identifying 12 significantly differentially expressed genes. By comparing all the candidate genes with the 168 genes identified in this study, the *CubHLH30* gene was selected. Previous studies have shown that *bHLH30* is one of the key transcription factors involved in anthocyanin biosynthesis and is closely related to the synthesis of cyanidin-3-O-galactoside, which is the main pigment responsible for red coloration in fruits. *bHLH30* promotes the synthesis of anthocyanins associated with color expression by regulating the expression of structural genes (Ye et al., 2023).

Furthermore, we found that *CubHLH17* also clustered within the IIIf subfamily based on the phylogenetic tree constructed using *A. thaliana* and motif prediction, which has been shown to be closely associated with anthocyanin biosynthesis in other species. For example, three candidate *ZjbHLH* genes in *Ziziphus jujuba* belong to the III subfamily and are highly related to anthocyanin biosynthesis (Shi et al., 2019). In *Cinnamomum camphora*, *CcbHH001*, *CcbHH022*, *CcbHH118*, and *CcbHH134* are classified into the IIIf subfamily and are homologous to *AtbHLH042*, which is associated with anthocyanin biosynthesis (Gong et al., 2023). The *SmbHLH1* and *SmbHLH117* genes in *Solanum melongena* have been shown to be highly related to anthocyanin biosynthesis (Tian et al., 2019). *CmbHLH2* has been confirmed to be involved in anthocyanin synthesis in *Chrysanthemum morifolium* (Xiang et al., 2015). Similarly, the VuMYB90-2, VuMYB90-3, VuMYB4-40, VuCPC, VuMYB, bHLH, and WD proteins of *Vigna unguiculata*, coordinate the regulation of anthocyanin and flavonoid accumulation (Li et al., 2020). In addition, a protein interaction network was constructed with *A. thaliana*, and *CubHLH17* was found to be most closely associated with *bHLH30*. To verify whether the *CubHLH17* gene has a regulatory role similar to that of *bHLH30*, we used qRT-PCR to verify the expression of *CubHLH17*. The results showed that the *CubHLH17* gene was significantly different between the Rsh and Gsh samples (**Figure 8B**), indicating that this gene may be related to the development of red bamboo shoot color and may play a key role in anthocyanin regulation, which provides the necessary transcription factor background for color-related biosynthesis pathways.

### 4.2 Transcription factors related to pigment biosynthesis

Pigment synthesis and accumulation are complex biological processes that involve the coordinated regulation of multiple genes, transcription factors, and environmental factors. Although the *CubHLH17* gene was stably expressed in all samples and significantly expressed in the red bamboo sheath, its ultimate function may not be produced independently, and it may require interaction with other regulatory factors. bHLH transcription factors typically form regulatory complexes with other transcription factors, such as MYB and WD40 repeat proteins, which affect the synthesis and accumulation of pigments through synergistic effects (Gonzalez et al., 2007; Xu et al., 2015; Chen et al., 2021b). Hence, we speculate that *CubHLH17* has an important influence on the development of bamboo shoot color through interactions with other factors. The stable expression of *CubHLH17* in samples of different colors indicates that it may be an essential component for maintaining normal operation of the pigment synthesis pathway. In the future, with the improvement in bamboo genome big data, we can further explore the interactions between *CubHLH17* and other key transcription factors and provide a new perspective for revealing the molecular regulatory network of bamboo sheath color changes through multilevel functional studies. We can also use plant genome editing tools such as the CRISPR-Cas9 system (Wu et al., 2024), RNA interference (Niu et al., 2008; Spada et al., 2024), and artificial microRNA technologies (Zhang et al., 2023) to target *CubHLH17* and transform it into a bamboo variety with potential industrial application value in terms of aesthetics, quality, and market value, thereby contributing to scientific research and commercial bamboo cultivation. In summary, the discovery and identification of *CubHLH17* contributes to a deeper understanding of the color variation of bamboo sheaths and provides important genetic insights for the improvement and industrial application of the bamboo sheath. We believe that this research has the potential to improve the color and quality of bamboo through gene editing, thereby driving innovation and progress in the bamboo industry.

## Data Availability

The datasets presented in this study can be found in online repositories. The names of the repository/repositories and accession number(s) can be found in the article/**Supplementary Material**.

## Supplementary material

Supplementary Figure 1 Heatmap of pairwise correlations between different samples or variables. Both the horizontal and vertical axes represent individual samples/variables. Blue indicates low correlation (close to 0.5) and red indicates high correlation (close to 1.0). The value in each cell represents the correlation coefficient between individual samples or variables. The dendrograms on the top and left show hierarchical clustering based on correlation features, with shorter branch lengths indicating higher similarity between samples.

Supplementary Figure 2 The secondary structure prediction of CubHLH proteins. Listed sequentially from top to bottom as CubHLH1 to CubHLH44.
