# Supplementary figures and images for: Functional characterization in Chimonobambusa utilis reveals the role of bHLH gene family in bamboo sheath color variation
Source: Front Plant Sci. 2025 Feb 12;16:1514703. doi: 10.3389/fpls.2025.1514703 (PMC11861543; doi:10.3389/fpls.2025.1514703)

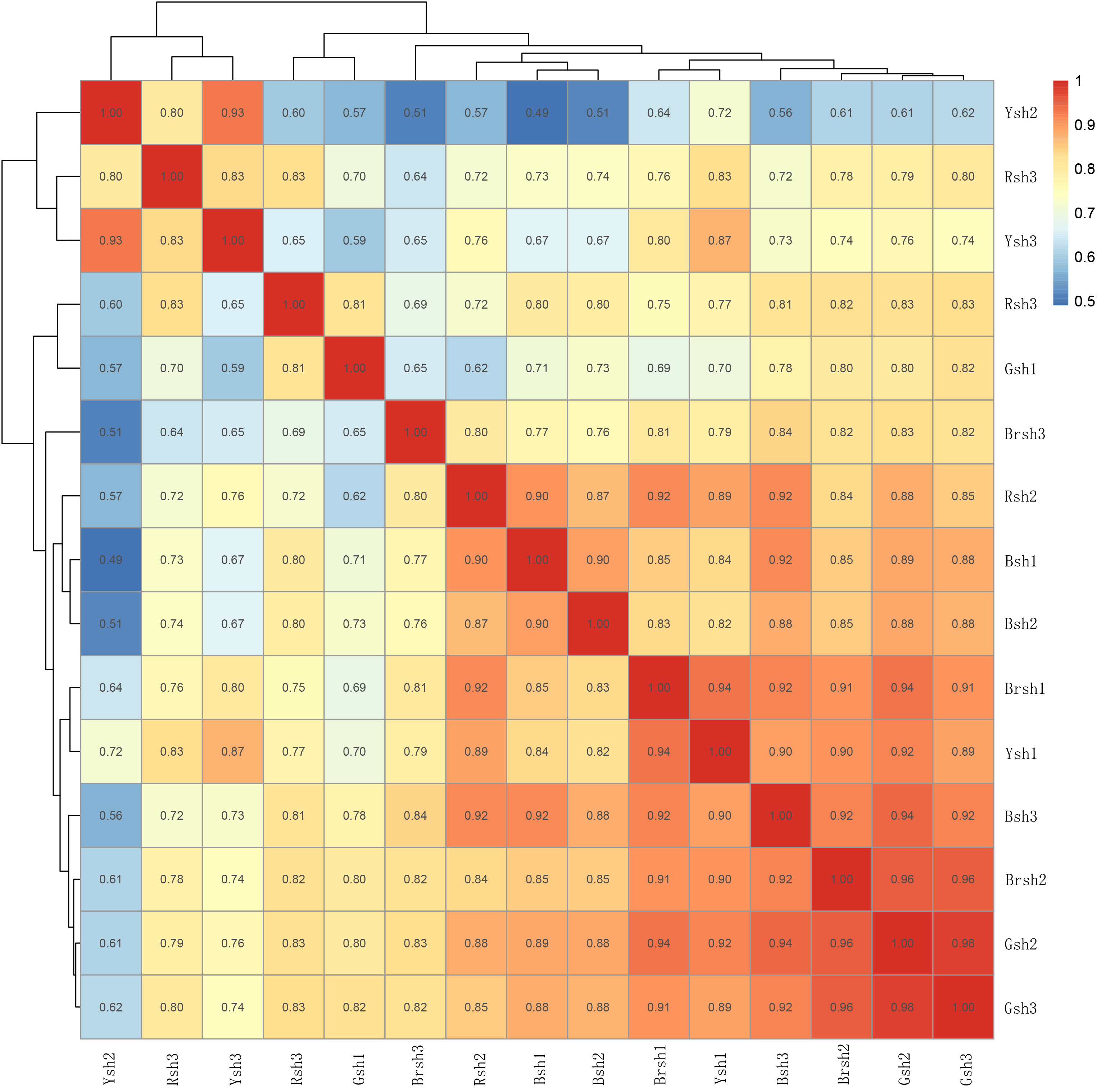

Supplement: Supplementary Figure 1 — Heatmap of pairwise correlations between different samples or variables. Both the horizontal and vertical axes represent individual samples/variables. Blue indicates low correlation (close to 0.5) and red indicates high correlation (close to 1.0). The value in each cell represents the correlation coefficient between individual samples or variables. The dendrograms on the top and left show hierarchical clustering based on correlation features, with shorter branch lengths indicating higher similarity between samples. [file Image1.jpeg]
