# Supplementary material for: Functional characterization in Chimonobambusa utilis reveals the role of bHLH gene family in bamboo sheath color variation
Source: Front Plant Sci. 2025 Feb 12;16:1514703. doi: 10.3389/fpls.2025.1514703 (PMC11861543; doi:10.3389/fpls.2025.1514703)
Supplement: Supplementary Figure 2 — The secondary structure prediction of CubHLH proteins. Listed sequentially from top to bottom as CubHLH1 to CubHLH44. [file DataSheet1.pdf]

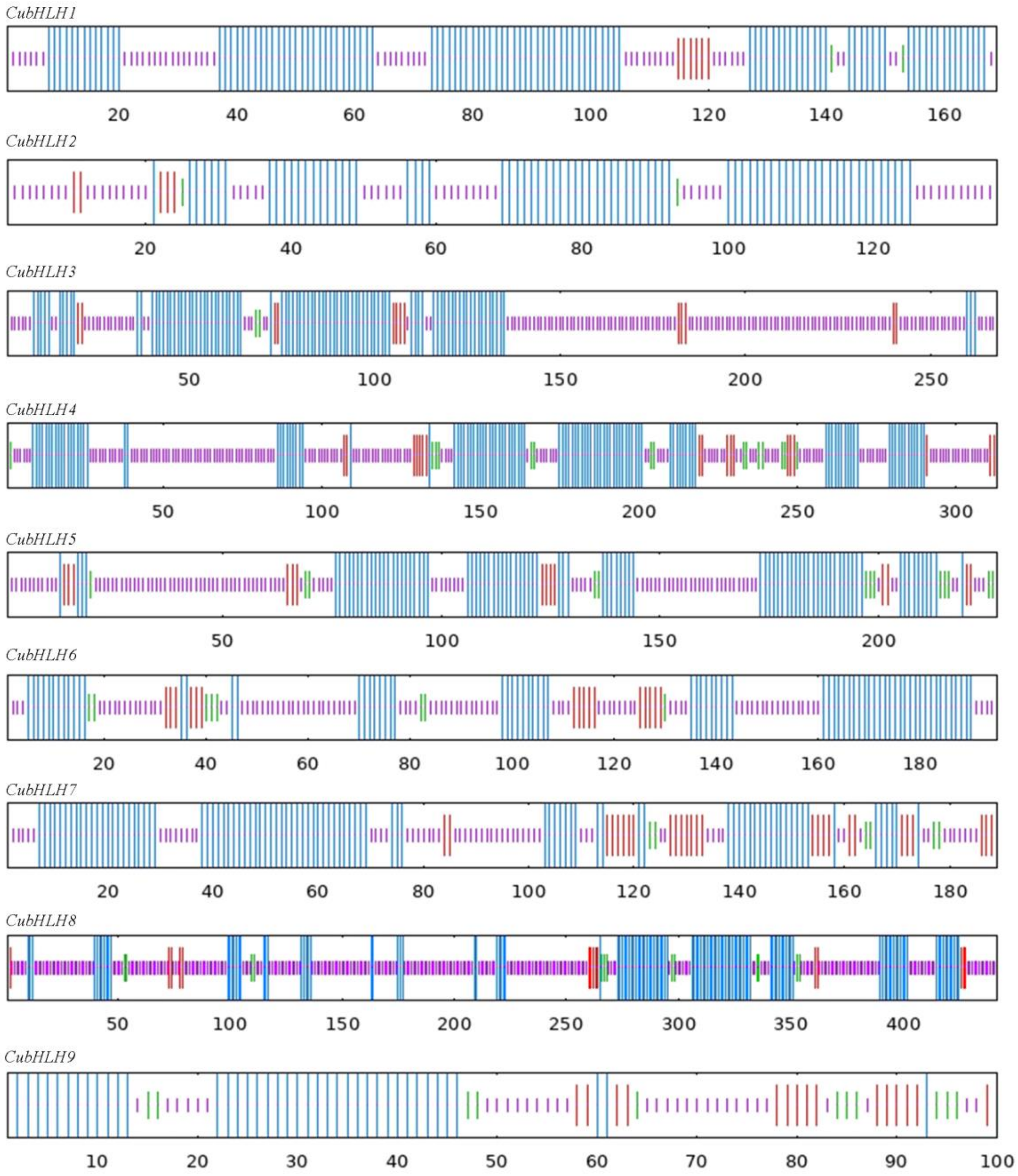
CubHLH1
20 40 60 80 100 120 140 160
CubHLH2
20 40 60 80 100 120
CubHLH3
50 100 150 200 250
CubHLH4
50 100 150 200 250 300
CubHLH5
50 100 150 200
CubHLH6
20 40 60 80 100 120 140 160 180
CubHLH7
20 40 60 80 100 120 140 160 180
CubHLH8
50 100 150 200 250 300 350 400
CubHLH9
10 20 30 40 50 60 70 80 90 100

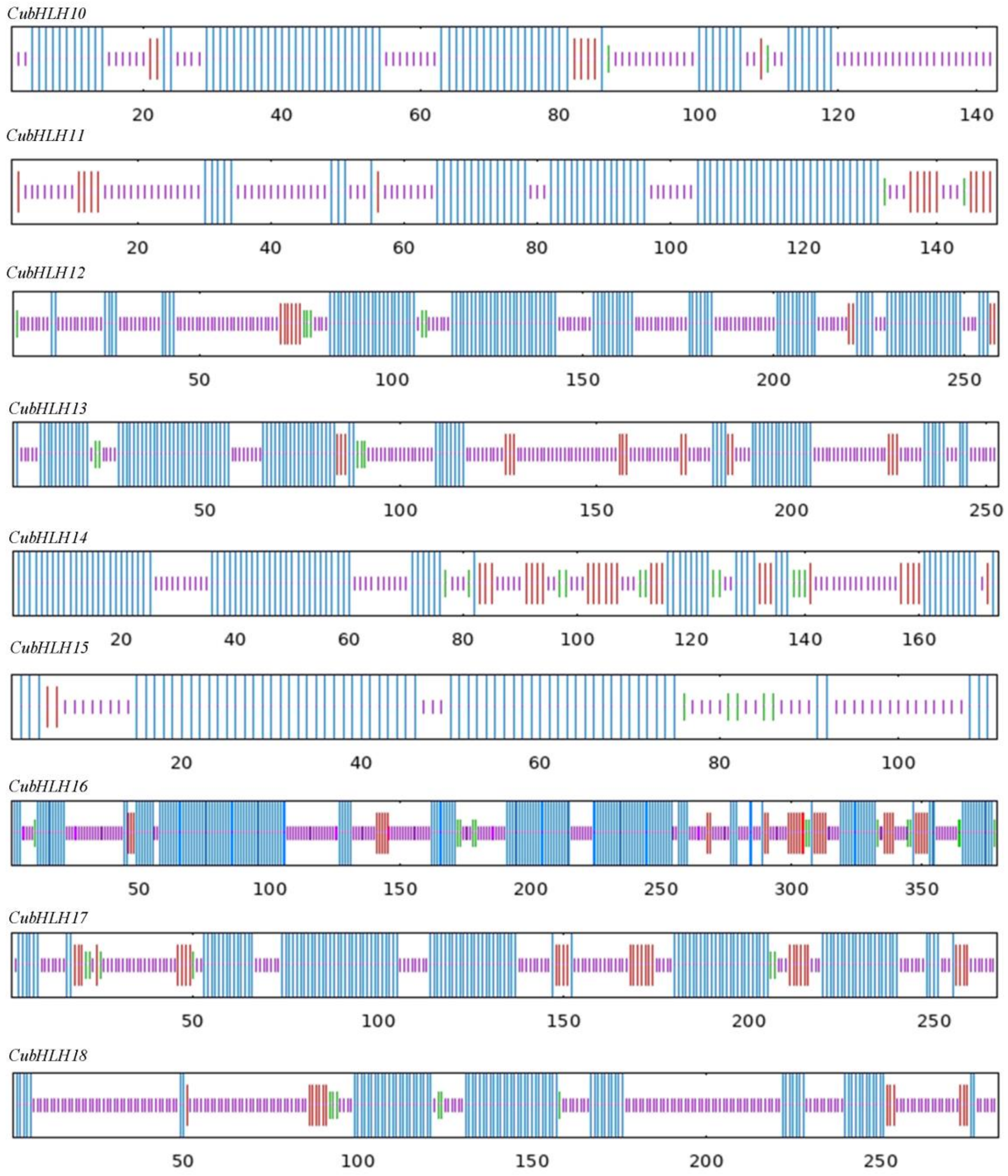
CubHLH10
20 40 60 80 100 120 140
CubHLH11
20 40 60 80 100 120 140
CubHLH12
50 100 150 200 250
CubHLH13
50 100 150 200 250
CubHLH14
20 40 60 80 100 120 140 160
CubHLH15
20 40 60 80 100
CubHLH16
50 100 150 200 250 300 350
CubHLH17
50 100 150 200 250
CubHLH18
50 100 150 200 250

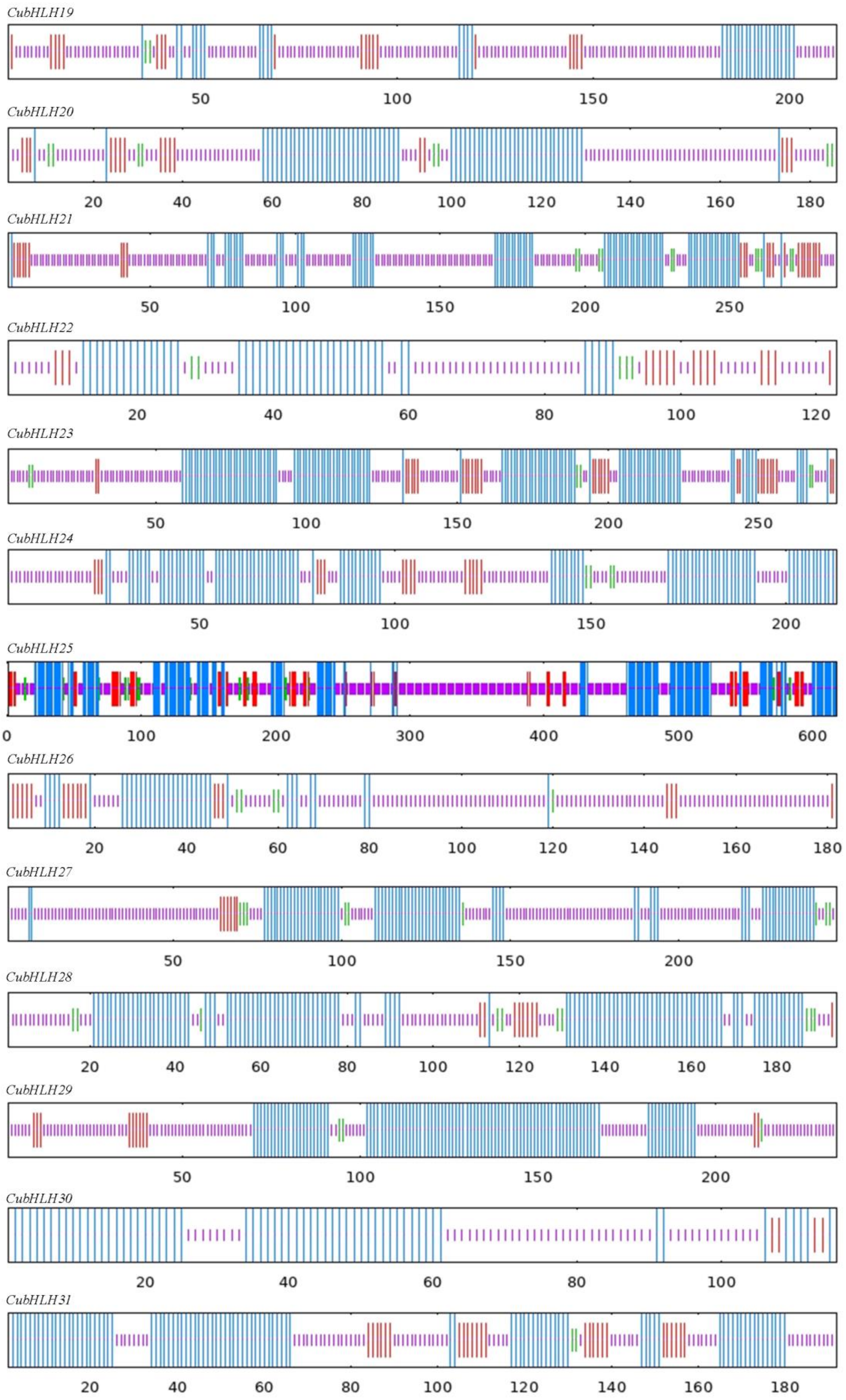
CubHLH19
50 100 150 200
CubHLH20
20 40 60 80 100 120 140 160 180
CubHLH21
50 100 150 200 250
CubHLH22
20 40 60 80 100 120
CubHLH23
50 100 150 200 250
CubHLH24
50 100 150 200
CubHLH25
0 100 200 300 400 500 600
CubHLH26
20 40 60 80 100 120 140 160 180
CubHLH27
50 100 150 200
CubHLH28
20 40 60 80 100 120 140 160 180
CubHLH29
50 100 150 200
CubHLH30
20 40 60 80 100
CubHLH31
20 40 60 80 100 120 140 160 180

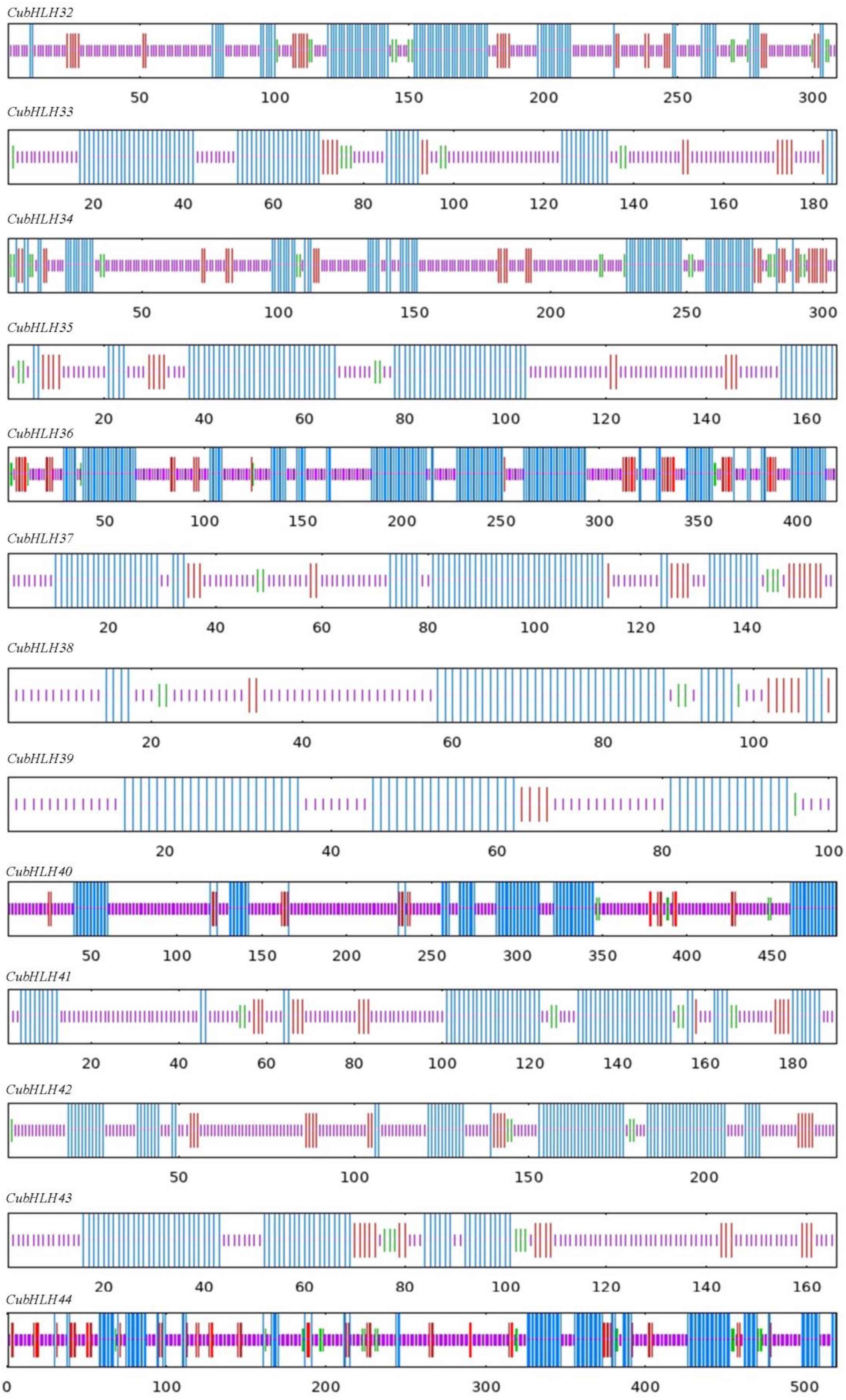
CubHLH32
50 100 150 200 250 300
CubHLH33
20 40 60 80 100 120 140 160 180
CubHLH34
50 100 150 200 250 300
CubHLH35
20 40 60 80 100 120 140 160
CubHLH36
50 100 150 200 250 300 350 400
CubHLH37
20 40 60 80 100 120 140
CubHLH38
20 40 60 80 100
CubHLH39
20 40 60 80 100
CubHLH40
50 100 150 200 250 300 350 400 450
CubHLH41
20 40 60 80 100 120 140 160 180
CubHLH42
50 100 150 200
CubHLH43
20 40 60 80 100 120 140 160
CubHLH44
0 100 200 300 400 500
