# Supplementary material for: Functional characterization in Chimonobambusa utilis reveals the role of bHLH gene family in bamboo sheath color variation
Source: Front Plant Sci. 2025 Feb 12;16:1514703. doi: 10.3389/fpls.2025.1514703 (PMC11861543; doi:10.3389/fpls.2025.1514703)
Supplement: Supplementary file 3 [file Table1.docx]

Supplementary Material

**
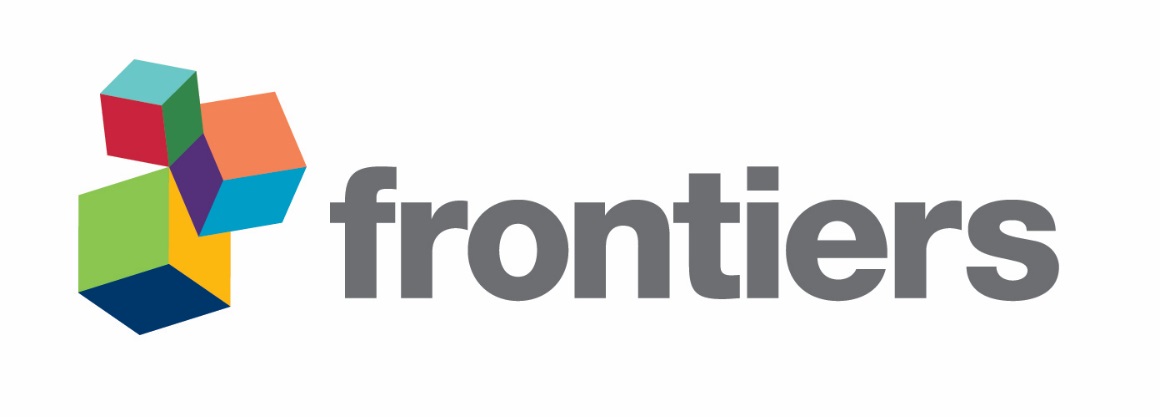
**

Table S1 The information of qRT-PCR primers

| Gene Name | F (Primer Sequence (5'-3')) | R (Primer Sequence (5'-3')) | Length (bp) |
| --- | --- | --- | --- |
| CuBHLH17 | TCTCAAGCTGAAGGTCGTCG | AGGCGGAGTTGACCTTATCG | 804 |
| TIP41-P | AAAATCATTGTAGGCCATTGTCG | ACTAAATTAAGCCAGCGGGAGTG | 102 |
