# Supplementary material for: Functional characterization in Chimonobambusa utilis reveals the role of bHLH gene family in bamboo sheath color variation
Source: Front Plant Sci. 2025 Feb 12;16:1514703. doi: 10.3389/fpls.2025.1514703 (PMC11861543; doi:10.3389/fpls.2025.1514703)
Supplement: Supplementary file 4 [file Table2.docx]

Supplementary Material

**
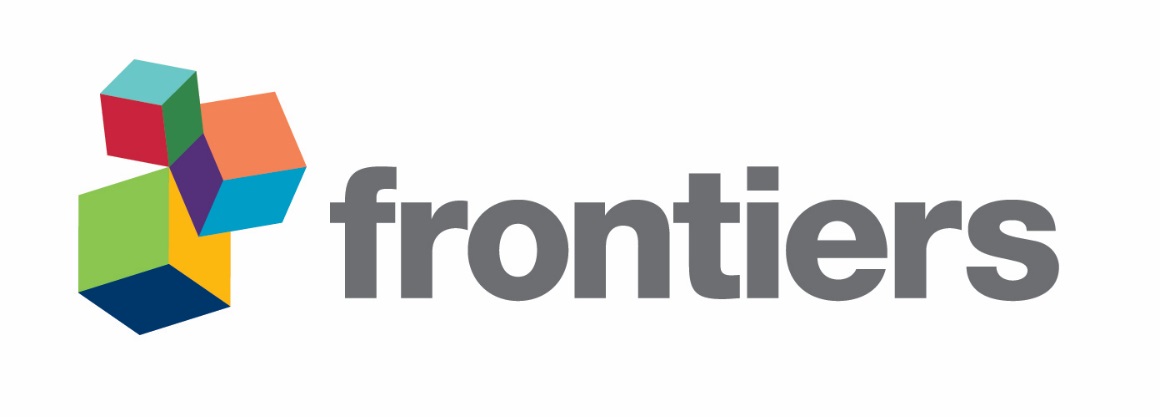
**

Table S2 Statistical information of the sample quality

| Sample | ReadSum | BaseSum | GC (%) | N (%) | Q20(%) | CycleQ20(%) | Q30(%) |
| --- | --- | --- | --- | --- | --- | --- | --- |
| Bsh1 | 29245458 | 8773637400 | 46.48 | 0 | 97.18 | 100 | 92.15 |
| Bsh 2 | 28669132 | 8600739600 | 46.14 | 0 | 97.05 | 100 | 91.84 |
| Bsh 3 | 31377098 | 9413129400 | 46.08 | 0 | 97.22 | 100 | 92.18 |
| Brsh1 | 25597174 | 7679152200 | 47.78 | 0 | 97.39 | 100 | 92.52 |
| Brsh2 | 28870945 | 8661283500 | 46.33 | 0 | 97.4 | 100 | 92.61 |
| Brsh3 | 30699221 | 9209766300 | 46.19 | 0 | 96.94 | 100 | 91.55 |
| Rsh1 | 31834262 | 9550278600 | 46.7 | 0 | 97.33 | 100 | 92.41 |
| Rsh2 | 31120060 | 9336018000 | 47.51 | 0 | 96.91 | 100 | 91.54 |
| Rsh3 | 27662766 | 8298829800 | 48.19 | 0 | 96.78 | 100 | 91.27 |
| Gsh1 | 28368438 | 8510531400 | 45.79 | 0 | 97.26 | 100 | 92.22 |
| Gsh2 | 28377358 | 8513207400 | 46.94 | 0 | 97.18 | 100 | 92.09 |
| Gsh3 | 28935002 | 8680500600 | 46.53 | 0 | 97.13 | 100 | 92.01 |
| Ysh1 | 30663472 | 9199041600 | 47.74 | 0 | 97.1 | 100 | 91.96 |
| Ysh2 | 28161643 | 8448492900 | 48.83 | 0 | 96.85 | 100 | 91.5 |
| Ysh3 | 28356578 | 8506973400 | 48.99 | 0 | 96.99 | 100 | 91.75 |
