# Supplementary material for: Functional characterization in Chimonobambusa utilis reveals the role of bHLH gene family in bamboo sheath color variation
Source: Front Plant Sci. 2025 Feb 12;16:1514703. doi: 10.3389/fpls.2025.1514703 (PMC11861543; doi:10.3389/fpls.2025.1514703)
Supplement: Supplementary file 5 [file Table3.docx]

Supplementary Material

**
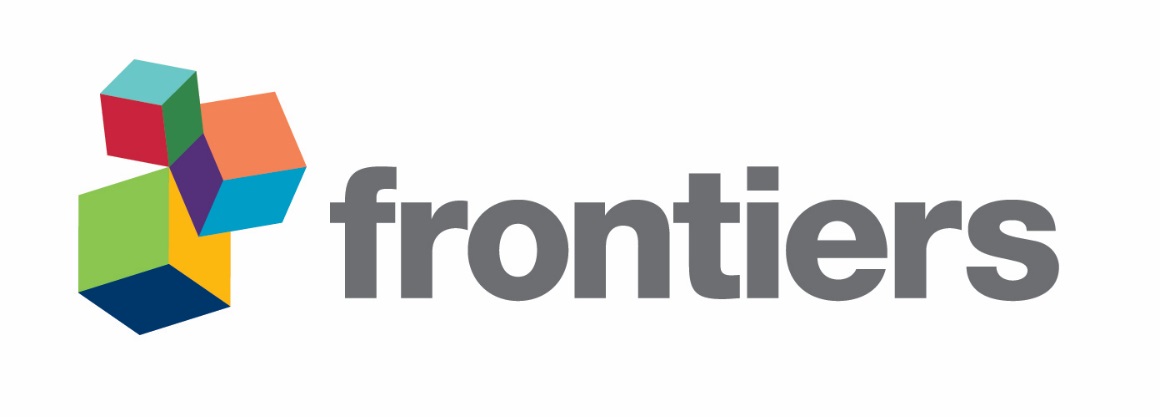
**

Table S3 Information of CuBHLH gene family members

| rename | Number of Amino Acid | Molecular Weight | Theoretical pI | Instability Index | Aliphatic Index | Grand Average of Hydropathicity | Predicted location(s) |
| --- | --- | --- | --- | --- | --- | --- | --- |
| CuBHLH1 | 169 | 18978.67 | 10.22 | 53.99 | 74.38 | -0.767 | Nucleus |
| CuBHLH2 | 137 | 15191.29 | 8.42 | 65.63 | 66.2 | -0.626 | Nucleus |
| CuBHLH3 | 268 | 29577.07 | 5.93 | 49.25 | 70.63 | -0.751 | Nucleus |
| CuBHLH4 | 313 | 33664.76 | 7.8 | 59.7 | 66.45 | -0.662 | Nucleus |
| CuBHLH5 | 227 | 24671.19 | 6.53 | 55.6 | 91.15 | -0.309 | Nucleus |
| CuBHLH6 | 195 | 21098.25 | 5.63 | 66.59 | 85.03 | -0.195 | Nucleus |
| CuBHLH7 | 189 | 21095.34 | 8.82 | 61.65 | 103.7 | -0.17 | Nucleus |
| CuBHLH8 | 442 | 47380.71 | 5.88 | 41.49 | 60.97 | -0.608 | Nucleus |
| CuBHLH9 | 100 | 10777.15 | 9.52 | 34.12 | 78 | -0.453 | Nucleus |
| CuBHLH10 | 143 | 15942.53 | 9.57 | 79.09 | 68.32 | -0.427 | Nucleus |
| CuBHLH11 | 149 | 16446.53 | 9.92 | 85.12 | 72.21 | -0.693 | Nucleus |
| CuBHLH12 | 259 | 28734.82 | 5.17 | 58.86 | 64.32 | -0.807 | Nucleus |
| CuBHLH13 | 253 | 27909.56 | 8.93 | 72.87 | 70.24 | -0.608 | Nucleus |
| CuBHLH14 | 174 | 19981.08 | 5.94 | 63.36 | 94.6 | -0.301 | Nucleus |
| CuBHLH15 | 111 | 12225.65 | 8.11 | 68.66 | 59.01 | -0.105 | Nucleus |
| CuBHLH16 | 379 | 41920.07 | 4.94 | 52.37 | 83.11 | -0.416 | Nucleus |
| CuBHLH17 | 267 | 28686.62 | 8.75 | 51.67 | 76.4 | -0.34 | Nucleus |
| CuBHLH19 | 284 | 31395.25 | 7.61 | 54.7 | 64.26 | -0.764 | Nucleus |
| CuBHLH18 | 212 | 22815.06 | 8.02 | 46.55 | 64.53 | -0.803 | Nucleus |
| CuBHLH20 | 186 | 20638.89 | 7.45 | 70.68 | 52.96 | -0.852 | Nucleus |
| CuBHLH21 | 287 | 31994.62 | 6.46 | 57.05 | 59.55 | -0.83 | Nucleus |
| CuBHLH22 | 123 | 13456.38 | 9.37 | 35.96 | 78.46 | -0.327 | Nucleus |
| CuBHLH23 | 276 | 30494.92 | 9.04 | 59.58 | 80.25 | -0.45 | Nucleus |
| CuBHLH24 | 213 | 23133.62 | 4.61 | 61.23 | 80.19 | -0.384 | Nucleus |
| CuBHLH25 | 618 | 67522.22 | 8.13 | 44.06 | 74.5 | -0.494 | Nucleus |
| CuBHLH26 | 182 | 20046.08 | 5.57 | 54.61 | 70.77 | -0.032 | Nucleus |
| CuBHLH27 | 247 | 26590.97 | 8.39 | 44.53 | 68.02 | -0.591 | Nucleus |
| CuBHLH28 | 194 | 21517.49 | 7.63 | 64.33 | 84.9 | -0.466 | Nucleus |
| CuBHLH29 | 234 | 25593.05 | 8.37 | 53.33 | 69.79 | -0.566 | Nucleus |
| CuBHLH30 | 116 | 12878.86 | 9.66 | 72.79 | 79.91 | -0.494 | Nucleus |
| CuBHLH31 | 192 | 21036.86 | 7.93 | 48.75 | 91.93 | -0.418 | Nucleus |
| CuBHLH32 | 309 | 33763.88 | 6.31 | 43.57 | 68.28 | -0.47 | Nucleus |
| CuBHLH33 | 185 | 21387.23 | 9.37 | 88.18 | 66.43 | -0.895 | Nucleus |
| CuBHLH34 | 305 | 33289.99 | 4.87 | 60.89 | 70.75 | -0.537 | Nucleus |
| CuBHLH35 | 166 | 18395.85 | 9.99 | 76.98 | 70.6 | -0.631 | Nucleus |
| CuBHLH36 | 420 | 46213.91 | 7.27 | 56.57 | 70.88 | -0.621 | Nucleus |
| CuBHLH37 | 157 | 17576.21 | 9.17 | 66.42 | 73.31 | -0.388 | Chloroplast |
| CuBHLH38 | 111 | 12446.25 | 10.46 | 77.31 | 78.29 | -0.68 | Nucleus |
| CuBHLH39 | 101 | 11125.96 | 9.66 | 41.45 | 97.52 | -0.383 | Nucleus |
| CuBHLH40 | 190 | 21152.78 | 9.02 | 59.43 | 77.58 | -0.739 | Nucleus |
| CuBHLH41 | 488 | 52160.66 | 6.17 | 59.77 | 66.72 | -0.515 | Nucleus |
| CuBHLH42 | 238 | 26237.46 | 8.45 | 51.5 | 65.21 | -0.494 | Nucleus |
| CuBHLH43 | 166 | 18091.44 | 7.2 | 83.7 | 70.54 | -0.581 | Nucleus |
| CuBHLH44 | 520 | 54787.2 | 5.11 | 55.75 | 74.9 | -0.326 | Nucleus |
