# Supplementary material for: Functional characterization in Chimonobambusa utilis reveals the role of bHLH gene family in bamboo sheath color variation
Source: Front Plant Sci. 2025 Feb 12;16:1514703. doi: 10.3389/fpls.2025.1514703 (PMC11861543; doi:10.3389/fpls.2025.1514703)
Supplement: Supplementary file 6 [file Table4.docx]

Supplementary Material

**
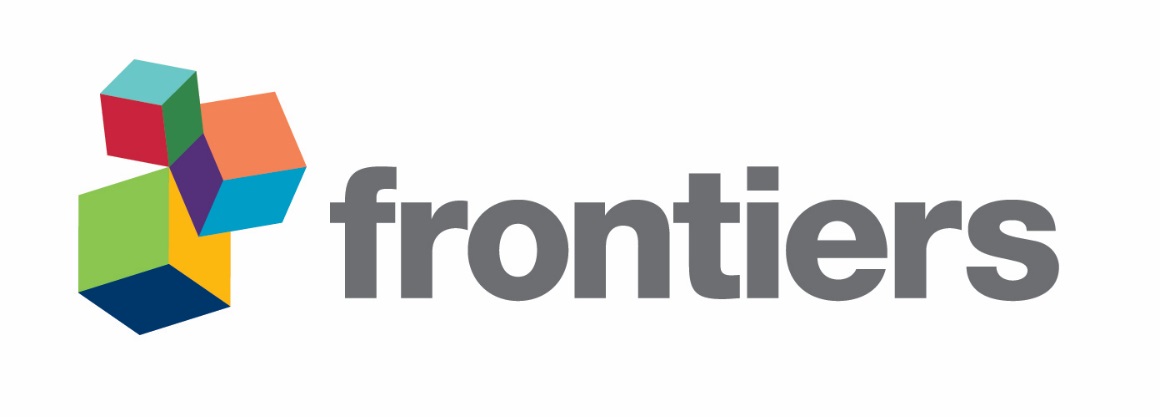
**

Table S4 Original ID correspondence of CubHLH gene family members

| Sequence ID | rename |
| --- | --- |
| TRINITY_DN10077_c0_g1.p1 | CubHLH1 |
| TRINITY_DN10315_c0_g1.p1 | CubHLH2 |
| TRINITY_DN11604_c0_g1.p1 | CubHLH3 |
| TRINITY_DN11934_c0_g1.p1 | CubHLH4 |
| TRINITY_DN1207_c0_g1.p1 | CubHLH5 |
| TRINITY_DN12470_c0_g1.p1 | CubHLH6 |
| TRINITY_DN12518_c0_g1.p2 | CubHLH7 |
| TRINITY_DN12819_c0_g1.p1 | CubHLH8 |
| TRINITY_DN13611_c0_g1.p1 | CubHLH9 |
| TRINITY_DN14687_c0_g1.p1 | CubHLH10 |
| TRINITY_DN16232_c0_g1.p2 | CubHLH11 |
| TRINITY_DN19548_c1_g1.p1 | CubHLH12 |
| TRINITY_DN2019_c0_g1.p1 | CubHLH13 |
| TRINITY_DN21446_c0_g1.p2 | CubHLH14 |
| TRINITY_DN22872_c0_g1.p1 | CubHLH15 |
| TRINITY_DN24368_c0_g1.p1 | CubHLH16 |
| TRINITY_DN25685_c0_g1.p1 | CubHLH17 |
| TRINITY_DN26137_c0_g1.p1 | CubHLH19 |
| TRINITY_DN261_c0_g1.p1 | CubHLH18 |
| TRINITY_DN26298_c0_g2.p2 | CubHLH20 |
| TRINITY_DN28280_c0_g1.p1 | CubHLH21 |
| TRINITY_DN28281_c0_g1.p1 | CubHLH22 |
| TRINITY_DN2875_c0_g1.p1 | CubHLH23 |
| TRINITY_DN29891_c0_g1.p1 | CubHLH24 |
| TRINITY_DN3180_c0_g1.p1 | CubHLH25 |
| TRINITY_DN33604_c0_g1.p1 | CubHLH26 |
| TRINITY_DN34063_c0_g1.p1 | CubHLH27 |
| TRINITY_DN35169_c0_g1.p1 | CubHLH28 |
| TRINITY_DN3579_c0_g1.p1 | CubHLH29 |
| TRINITY_DN36491_c0_g1.p2 | CubHLH30 |
| TRINITY_DN36779_c0_g2.p1 | CubHLH31 |
| TRINITY_DN42477_c0_g1.p1 | CubHLH32 |
| TRINITY_DN44235_c0_g1.p1 | CubHLH33 |
| TRINITY_DN45323_c0_g2.p1 | CubHLH34 |
| TRINITY_DN45422_c0_g1.p2 | CubHLH35 |
| TRINITY_DN50700_c0_g1.p1 | CubHLH36 |
| TRINITY_DN5229_c0_g1.p2 | CubHLH37 |
| TRINITY_DN5559_c0_g1.p3 | CubHLH38 |
| TRINITY_DN56191_c0_g1.p1 | CubHLH39 |
| TRINITY_DN573_c0_g1.p1 | CubHLH40 |
| TRINITY_DN57380_c0_g1.p1 | CubHLH41 |
| TRINITY_DN6570_c0_g1.p1 | CubHLH42 |
| TRINITY_DN8114_c0_g1.p1 | CubHLH43 |
| TRINITY_DN8164_c0_g1.p1 | CubHLH44 |
