# Supplementary material for: Functional characterization in Chimonobambusa utilis reveals the role of bHLH gene family in bamboo sheath color variation
Source: Front Plant Sci. 2025 Feb 12;16:1514703. doi: 10.3389/fpls.2025.1514703 (PMC11861543; doi:10.3389/fpls.2025.1514703)
Supplement: Supplementary file 9 [file Table7.docx]

Supplementary Material

**
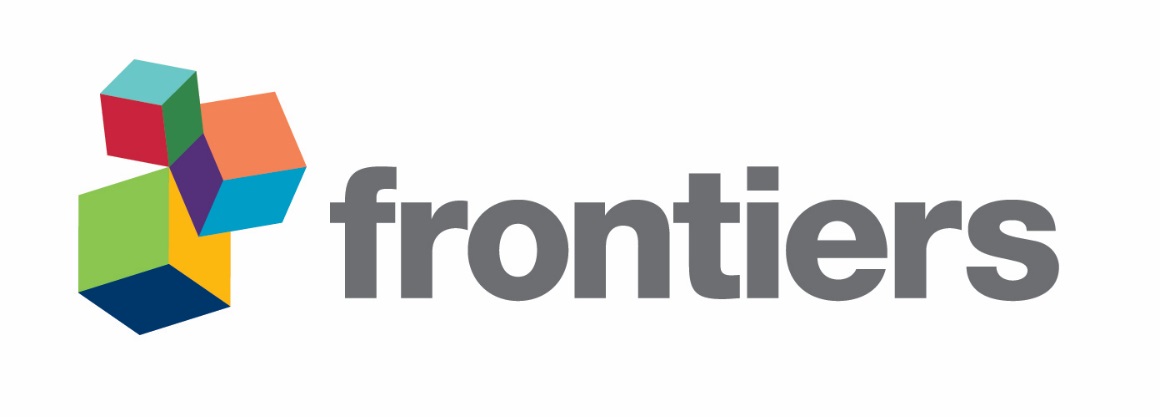
**

Table S7 Differential analysis results of CubHLHs

| Comparison | Up-regulated Genes | Down-regulated Genes |
| --- | --- | --- |
| Bsh vs Brsh | *CuBHLH4* | *CuBHLH7*, *CuBHLH1*, *CuBHLH30* |
| Bsh vs Rsh | *CuBHLH44*, *CuBHLH4*, *CuBHLH18* | *CuBHLH7*, *CuBHLH31*, *CuBHLH1* |
| Bsh vs Rsh | *CuBHLH4*, *CuBHLH3* | *CuBHLH7*, *CuBHLH41*, *CuBHLH1* |
| Bsh vs Ysh | *CuBHLH27*, *CuBHLH44* | *CuBHLH7*, *CuBHLH30*, *CuBHLH31*, *CuBHLH2*, *CuBHLH22* |
| Brsh vs Gsh | *CuBHLH3*, *CuBHLH30* | *CuBHLH41* |
| Rsh vs Brsh | *CuBHLH31* | *CuBHLH18*, *CuBHLH30* |
| Rsh vs Gsh | *CuBHLH31*, *CuBHLH3* | *CuBHLH18*, *CuBHLH41* |
| Rsh vs Ysh | None | *CuBHLH18*, *CuBHLH30* |
| Ysh vs Brsh | None | None |
| Ysh vs Gsh | *CuBHLH4*, *CuBHLH3*, *CuBHLH30* | *CuBHLH41* |
